# Triclocarban exposure at environmentally relevant concentrations perturbs the gut microbiota and metabolic profile in *Rana taihangensis* (Anura, Ranidae) tadpoles

**DOI:** 10.3389/fmicb.2025.1740880

**Published:** 2025-12-15

**Authors:** Ruinan Zhao, Pengyuan Liu, Yaoyang Wu, Hongyan Bi, Jiaoying He, Jie Zhang, Yanfu Qu, Xiaohong Chen, Zhuo Chen

**Affiliations:** 1 The Observation and Research Field Station of Taihang Mountain Forest Ecosystems of Henan Province, College of Life Sciences, Henan Normal University, Xinxiang, China; 2 College of Fisheries, Henan Normal University, Xinxiang, China; 3 Herpetological Research Center, College of Life Sciences, Nanjing Normal University, Nanjing, Jiangsu, China; 4 Key Laboratory of Yellow River and Huai River Water Environment and Pollution Control, Ministry of Education, Henan Normal University, Xinxiang, China

**Keywords:** amphibian, *Rana taihangensis*, triclocarban, gut microbiota, metabolome

## Abstract

**Introduction:**

The antibacterial agent triclocarban (TCC) poses a significant threat to aquatic ecosystems and its impact on amphibians remain poorly understood.

**Methods:**

Here, we investigated its acute and chronic effects on Rana taihangensis tadpoles at environmentally relevant concentrations (i.e., 5, 15 and 45 μg/L) using an integrated approach combining morphology, gut microbiome, and non-targeted metabolomic analyses.

**Results and Discussion:**

The acute toxicity tests for 96 h revealed that TCC had a lethal concentration (LC50) value of 169.863 μg/L for R. taihangensis tadpoles. Chronic exposure resulted in reduced body condition score across all the three TCC-treated groups compared to the control. Gut microbiome analysis revealed that TCC exposure significantly altered the community composition at both phylum (e.g., Pseudomonadota and Fusobacteriota) and genus (e.g., *Cetobacterium and Citrobacter*) levels. In addition, several metabolites (e.g., 20-carboxy-leukotriene B4, 11b-PGF2a, and leukotriene E4) associated with immune response and neural signaling were significantly perturbed in TCC-exposed tadpoles. Interestingly, correlation analysis indicated a significant relationship between specific metabolite changes and shifts in gut microbiota. Overall, our findings demonstrated that TCC exposure adversely affects the growth indexes, gut microbial composition and metabolites in R. taihangensis tadpoles, and the present study will provide new insights into the ecotoxicological risks of TCC and enhance the understanding of its mechanisms of toxicity in aquatic organisms.

## 1 Introduction

Water pollution represents a major global environmental challenge, posing significant risks to both human health and aquatic ecosystems (Malaj et al., 2014; Arenas-Sánchez et al., 2016; Mushtaq et al., 2020). Based on their nature and origin, water pollutants can be categorized into physical, chemical, biological and emerging contaminants, etc. (Fawell and Nieuwenhuijsen, 2003; Ashbolt, 2015; Jambeck et al., 2015; Santhappan et al., 2024). Among emerging pollutants, TCC has been widely used since 1957 as a polychlorinated aromatic antibacterial agent in products such as detergents, cosmetics, personal care items, and medical disinfectants (Halden, 2014; Ye et al., 2016). The widespread application of TCC-containing products, coupled with its incomplete removal in wastewater treatment plants and the direct discharge of untreated sewage, has led to its pervasive presence in aquatic environments (Halden and Paull, 2005; Xia et al., 2010). Moreover, TCC exhibits considerable environmental persistence, with a reported half-life of 60 days in water and 540 days in sediment (Halden and Paull, 2005), leading to its gradual accumulation in the environment over time (Higgins et al., 2011). TCC has been detected in various water bodies worldwide, including an Urban stream in the United States (5.6 μg/L), in the Kaveri River in India (1.12 μg/L), and the Weishui River in China (47.37 μg/L) (Halden and Paull, 2004; Vimalkumar et al., 2018; Zou and Liu, 2019).

The potential threats of TCC to aquatic animals have attracted extensive attention, with numerous studies documenting its adverse effects across various species. For example, in zebrafish (*Danio rerio*), TCC exposure has been shown to impair embryonic development (Dong et al., 2018; Shi et al., 2019), morphological development (Caioni et al., 2021; Zhang Y. et al., 2023), and multiple physiological systems, including the immune (Wei et al., 2018; Zhou et al., 2019), visual (Chen et al., 2021; Caioni et al., 2023), digestive (Yan et al., 2022), endocrine (Shi et al., 2019; Caioni et al., 2021), and nervous systems (Phillips et al., 2022; Lucon-Xiccato et al., 2023). Additionally, TCC can weaken the olfactory ability of goldfish by inhibiting odor recognition efficiency, disrupting signal generation and conduction, and interfering with olfactory information processing (Huang L. et al., 2023). Negative responses in biochemical indicators and behavior have also been reported in the amphipod *Gammarus locusta* (Barros et al., 2017). Although considerable progress has been made in recent years toward advancing our understanding of the TCC toxicity in various aquatic organisms, its effects on amphibians (an ecologically vital group in aquatic ecosystems) remain poorly understood. During the tadpole stage, amphibians are fully aquatic and particularly vulnerable to aquatic pollutants due to their highly permeable and exposed skin (Alford et al., 2001; Chen J. Y. et al., 2022; Ding et al., 2022; Xie et al., 2024). This physiological trait makes them sensitive biological indicators for assessing the health and pollution levels of aquatic ecosystems.

Accumulating evidence indicated that environmental pollutants can significantly disrupt the intestinal microecological balance of tadpoles and affect host health (Lv et al., 2023; Zhang Q. et al., 2023; Zhu et al., 2023). The gastrointestinal tract is the important place for material exchange with the external environment and harbors diverse microbial communities (Takiishi et al., 2017; Yang et al., 2019). These gut microbes play important roles in digestion, nutrient absorption, metabolism, and the regulation of the immune function and pathogen defense (Dawood and Koshio, 2016; Gao et al., 2020). Studies have shown that exposure to environmental pollutants and antibiotics can disturb the composition and function of gut microorganisms, thereby affecting host health (Claus et al., 2016; Evariste et al., 2019). In addition, gut microbiota contributes to host metabolism and energy acquisition by producing essential enzymes that the host cannot synthesize (Rowland et al., 2018). Microbial metabolites, such as short-chain fatty acids, significantly influence host immune regulation (Chen et al., 2017). The diversity and stability of the gut microbiota are particularly crucial for hosts during key life stages such as amphibian metamorphosis (Colombo et al., 2015). Changes in the abundance of specific intestinal microbial groups can further affect host metabolic processes (Lamichhane et al., 2018). For instance, environmental pollutant exposure has been shown to induce key changes in intestinal metabolites and metabolic pathways in *Pelophylax nigromaculatus* tadpoles (Huang M. et al., 2023). Non-targeted metabolomics offers a powerful approach to evaluate the biological effects of environmental stressors at the molecular level (Dumas et al., 2020), with liquid chromatography-mass spectrometry (LC-MS) serving as a valuable technique for identifying biochemical changes and differential metabolism in biological liquids (Samuelsson and Larsson, 2008). Nevertheless, the potential toxicity and adverse effects of TCC exposure on the gut microbiota and metabolome of amphibians remain poorly understood.

*Rana taihangensis* (Anura, Ranidae, *Rana*) (Shen et al., 2022) could be one of the most ideal candidate frogs for studying the toxicological effects of TCC on amphibians. This is based on its native distribution and the documented vulnerability of its montane habitat to anthropogenic contaminants (Wang et al., 2024). Given that TCC is a prevalent environmental pollutant frequently detected in aquatic systems (Halden and Paull, 2005; Xia et al., 2010), resident species like *R. taihangensis* are at potential risk, warranting investigation into its specific effects. In this study, we investigated the acute toxicity and chronic effects of TCC at environmentally relevant concentrations (i.e., 5, 15 and 45 μg/L) on *R. taihangensis* tadpoles with the solvent group as control, using a combination of morphology, microbiome and non-targeted metabolomics. Our objectives were to (1) examine the effects of TCC exposure on the early developmental stages of *R. taihangensis*; (2) address the potential changes of the morphology, intestinal microbial community and endogenous metabolism caused by TCC exposure; (3) determine the key metabolic pathways affected by TCC and evaluate whether changes of the gut microbiota were associated with the changes in the metabolome. This study will provide new insights into the ecotoxicological risks of TCC and enhance the understanding of its mechanisms of toxicity in aquatic organisms.

## 2 Materials and methods

### 2.1 Chemicals and reagents

Triclocarban (TCC, 98% purity, CAS: 101-20-2) and dimethyl sulfoxide (DMSO, 99% purity, CAS: 67-68-5) were purchased from Aladdin (Shanghai, China).

### 2.2 Animals and treatments

Sexual maturity samples of *R. taihangensis* were collected from the Taihangshan Mountain in Huixian City, Henan province, China, in April 2023, and reared in the laboratory conditions until spawning. Then, the freshly laid eggs were transferred to aquatic containers (45 cm × 33 cm × 13 cm) filled with water to a depth of 4 cm and maintained at a water temperature of 21 ± 1 °C. A photoperiod of 12 h light to 12 h dark was maintained throughout the whole experiment. The aerated water used in the experiment had a pH of 8.14 ± 0.02, conductivity of 300.8 ± 3.31 μS/cm, and dissolved oxygen levels of 8.18 ± 0.10 mg/L (mean ± SD). Tadpoles were fed daily with spirulina dry powder, and water was renewed every other day. This laboratory rearing protocol from the embryonic stage ensured that all experimental tadpoles were naïve to TCC and other environmental pollutants prior to the commencement of the exposure experiments. The developmental stages of tadpoles were determined according to Gosner (1960). Once the tadpoles reached Gosner stage (Gs) 26–28, they were exposed to TCC. A stock solution of 0.5 g/L were prepared using DMSO, stored at 4 °C, and diluted as needed for exposure experiments. The final DMSO concentration in all test solutions, including the control, did not exceed 0.05 ‰ (v/v). Previous studies have demonstrated that 0.5‰ DMSO, with the concentration used in this experiment being much lower than 0.5‰, exerted no adverse effects on the survival, metamorphosis, body size parameters (e.g., body mass, snout-vent length) and hepatic physiological functions (e.g., hepatic somatic index, antioxidant enzyme activity) of *Hoplobatrachus rugulosus* tadpoles (Chen J. Y. et al., 2022). All animal sampling and usage protocols were implemented in compliance with all ethical guidelines and legal regulations in China and approved by the Institutional Animal Care and Ethics Committee of Henan Normal University (protocol code IACEC20230027 and March 24, 2023).

#### 2.2.1 Experiment 1: acute toxicity tests

A preliminary range-finding test was conducted over 96 h to estimate the median LC_50_ of TCC for tadpoles. Mortality was determined by the absence of movement after tactile stimulation of the head with a glass rod. Based on the results of preliminary experiments, TCC concentrations ranging from 100 to 600 μg/L were selected for the formal assay. Tadpoles at Gosner stage 26–28 were randomly distributed into 11 TCC exposure groups and one solvent control group. Each group was held in containers (29 cm × 21 cm × 9 cm) containing 2 L of aerated water, with 10 tadpoles per container and three replicates per treatment. The solvent control group received the same volume of DMSO as present in the 600 μg/L TCC exposure group. To maintain consistent water quality and stable TCC concentrations, the test solutions were renewed every 24 h. All other rearing conditions matched those described in the previous section. Tadpole mortality was recorded at 24, 48, 72, and 96 h after exposure, and the LC_50_ value was calculated accordingly.

#### 2.2.2 Experiment 2: chronic toxicity tests

To assess the chronic toxicity of TCC in *R. taihangensis*, tadpoles were exposed to three environmentally relevant TCC concentrations (5, 15, 45 μg/L), which are designed to form an equal-ratio concentration gradient (1:3:9), along with a solvent control group. The low concentration (TL, 5 μg/L) was set to mimic typical TCC levels in moderately polluted natural freshwater systems. This choice was based on a reported TCC concentration of 6.75 μg/L in the Maryland River (Halden and Paull, 2005). The medium concentration (TM, 15 μg/L) was designed to target sublethal chronic effects, following the classic toxicological practice of using 1/10 of the 96-h median lethal concentration (96 h-LC_50_). The high concentration (TH, 45 μg/L) was selected to simulate extreme TCC pollution scenarios in freshwater systems impacted by intense anthropogenic activity and it is based on the maximum reported environmental TCC concentration of 47.37 μg/L detected in the Weishui River, Changsha City, China (Zou and Liu, 2019). Three TCC stock solutions (100, 300, and 900 mg/L) were prepared by dissolving 10, 30, and 90 mg of TCC, respectively, in 100 mL of DMSO, and stored in the dark at 4 °C until use. All treatments, including the control, contained the same volume of DMSO to ensure consistency. Each treatment was conducted in triplicate, with 17 tadpoles per tank in 2 L of aerated water. Test solutions were renewed every 2 days, and rearing conditions matched those described for the acute toxicity tests. After 21 days of TCC exposure, tadpoles were euthanized using 100 mg/L MS-222. Body mass, total length and snout-vent length (SVL) were recorded. The body condition score was calculated as 100 × (body mass/body length^3^) (Zhang Q. et al., 2023). Intestinal guts (including gut contents) were collected for gut microbiota analysis, and whole tadpoles were sampled for metabolomic profiling.

### 2.3 Microbiota sequencing and data analysis

A total of 12 tadpoles were sampled from each concentration group for intestinal microbiota analysis. From each of the three replicate containers per group, four tadpoles were collected, and every two individuals were pooled to form one sample, resulting in six replicate samples per concentration group. Total genomic DNA was extracted using the E.Z.N.A.^®^ stool DNA Kit (Omega Bio-Tek, Norcross, GA, United States) according to the manufacturer’s instructions. DNA integrity and purity were evaluated via electrophoresis on a 1% agarose gel and quantified using a Qubit@3.0 fluorometer (Thermo Scientific), respectively. The V3–V4 hypervariable region of the bacterial 16S rRNA gene was amplified using the forward primer 338F (5′-ACTCCTACGGGAGGCAGCA-3′) and reverse primer 806R (5′-GGACTACHVGGGTWTCTAAT-3′). The PCR reaction mixture consisted of 30 μL, including 20 ng of DNA templates, 1 μL of each primer, and 15 μL of 2 × Taq Master Mix (TaKaRa, China). The PCR cycling protocol was as follows: initial denaturation at 98 °C for 5 min, followed by 25 cycles of 98 °C for 30 s, 52 °C for 30 s, and 72 °C for 45 s, with a final extension at 72 °C for 5 min. Pair-end 2 × 250 bp sequencing was performed on the Illumina NovaSeq platform at Shanghai Personal Biotechnology Co., Ltd. (Shanghai, China). Bioinformatics analysis was conducted using QIIME2 v2019.4 (Bolyen et al., 2019) and R packages (v3.2.0). Raw sequences were processed using the DADA2 v1.20 plugin (Callahan et al., 2016) to perform quality filtering, denoising, merging, chimera removal, resulting in amplicon sequence variants (ASVs). Taxonomic annotation was carried out against the SILVA 132 database (Quast et al., 2013). Alpha-diversity indices (Chao1 richness index, Simpson diversity index and Pielou’s evenness index) were calculated from the ASV table in QIIME2, and group differences were assessed using the Kruskal–Wallis test. Beta diversity was evaluated by principal coordinate analysis (PCoA) based on both weighted UniFrac and unweighted UniFrac distances using the Vegan package in R, and statistical significance among groups was tested using PERMANOVA (Adonis test with 999 permutations). Differential microbial taxa between the solvent control and TCC-treated groups were identified at the phylum and genus levels using the Kruskal–Wallis test followed by Dunn’s *post hoc* test. Additionally, biomarkers were identified using linear discriminant analysis effect size (LEfSe), with a linear discriminant analysis (LDA) threshold score of 2.0.

### 2.4 Metabolome analyses

For metabolomic analysis, each tadpole was treated as an independent sample and homogenized in 200 μL of ice-cold water using a high-throughput tissue grinder. A total of 24 samples were processed, comprising six biological replicates per treatment group across the four experimental groups. Metabolites were extracted by adding 800 μL of methanol-acetonitrile mixed solution (1:1, v/v), followed by ultrasonication for 30 min, incubation at −20 °C for 30 min, and centrifugation at 12,000 rpm for 10 min. The resulting supernatant was collected, concentrated under vacuum, and reconstituted in 150 μL of 50% methanol containing 5 ppm 2-chlorophenylalanine. After vortexing for 30 s and recentrifugation, 10–20 μL of supernatant from each sample was pooled into a quality control (QC) sample to assess instrumental stability and data reliability. Metabolite profiling was performed on an ACQUITY UPLC HSS T3 column (100 Å, 1.8 μm, 2.1 mm × 100 mm) and analyzed using a Thermo Orbital Trap Exploris 120 mass spectrometer controlled by Compound Discoverer^™^ v3.3.2.31 (Thermo, Waltham, United States). Orthogonal partial least squares discriminant analysis (OPLS-DA) was conducted using the Ropls package in R to reduce data dimensionality and visualize group separation. Differential expressed metabolites were identified based on a combination of univariate and multivariate criteria: *p*-values <0.05 (Student’s *t*-test), variable importance in projection (VIP) >1 from the OPLS-DA model, and fold change (FC). Model quality was assessed using *R*^2^*Y* (goodness of fit) and *Q*^2^ (predictive ability), with values closer to 1 indicating stronger model performance. Venn diagrams generated with the VennDiagram v1.7.3 package were used to illustrate common and unique metabolites across comparison groups. Shared differential metabolites identified across all TCC-treated groups were subjected to KEGG pathway enrichment analysis using the clusterProfiler package (v4.6.0). Metabolic pathways with *p*-value <0.05 and containing at least five differentially abundant metabolites were considered significantly enriched and further analyzed.

### 2.5 Conjoint analysis of microbial and metabolome

LEfSe was employed to identify potential microbial and metabolite markers, with a LDA score threshold set at 2.0. Spearman correlation analysis was then performed to evaluate the associations between the identified microbial and metabolite biomarkers. Only marker pairs showing strong correlations (|rho| >0.7) were retained for subsequent analysis. Additionally, bar plots illustrating differentially expressed metabolites between the solvent control group and the TCC-treated groups were generated using Origin software.

### 2.6 Statistical analyses

In the acute toxicity assays, probit analysis (Finney, 1978) was employed to estimate the LC_50_ (with a 95% confidence interval) using mortality data recorded at 24-h intervals. The Kolmogorov–Smirnov and Shapiro–Wilk tests were used to evaluate whether the data significantly deviated from a normal distribution, while Levene’s test was conducted to assess homoscedasticity across groups. As the data violated parametric assumptions, differences in morphological traits among groups were analyzed using the Kruskal–Wallis test, followed by Dunn’s *post hoc* test for multiple comparisons. All data are presented as mean ± standard error (SE), and statistical significance was set at *p* < 0.05.

## 3 Results

### 3.1 Effects of TCC exposure on acute toxicity and morphological indexes of tadpoles

**Figure 1:**
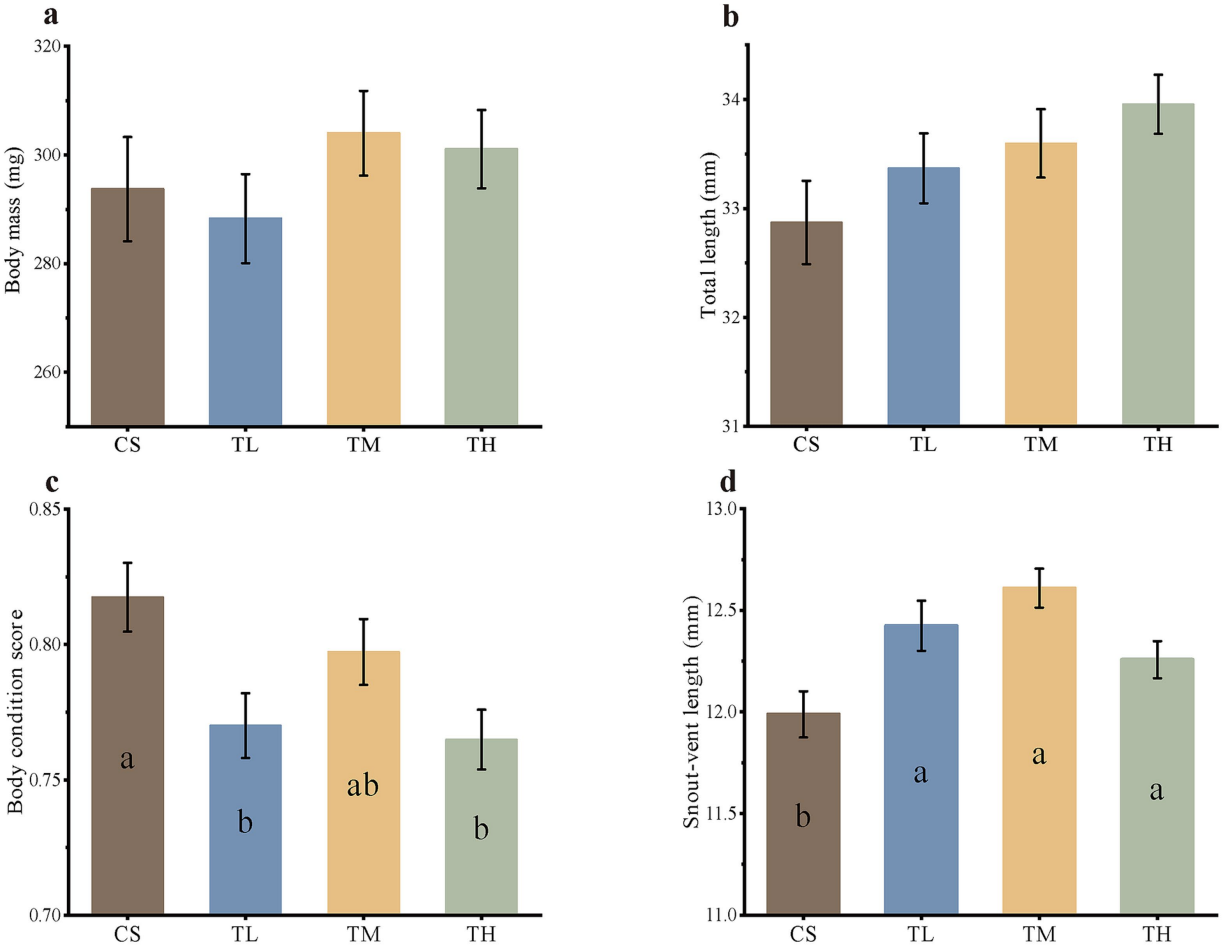
Morphological indexes of *R. taihangensis* tadpoles exposed to solvent control (CS), 5 μg/L (TL), 15 μg/L (TM), and 45 μg/L (TH) of TCC. Intergroup differences in body mass **(a)**, total length **(b)**, body condition score, **(c)** and snout-vent length **(d)** after 3 weeks of treatment. Each bar stands for mean ± SE, and different letters denote significant differences between groups (*p* < 0.05, Kruskal–Wallis test and Dunn’s *post hoc* test).

Probit regression analysis determined the median LC_50_ of TCC for *R. taihangensis* tadpoles to be 343.708, 228.342, 197.477, and 169.863 μg/L at 24, 48, 72, and 96 h, respectively (see Supplementary Table S1, Supplementary Figure S1, and Table 1 for detailed data). Morphological traits, including body mass, total length, SVL and body condition score, were compared in the solvent control and TCC-treated groups (5, 15, 45 μg/L), with results presented in Figure 1. While no statistically significant differences in body mass (*H* = 3.006, df = 3, *p* = 0.391) or total length (*H* = 5.874, df = 3, *p* = 0.118) were observed between TCC-exposed and control tadpoles, a non-significant increasing trend in total length was detected with TCC exposure (Figures 1a,b). In contrast, the body condition score was reduced in all three TCC treatment groups compared to the control (*H* = 11.093, df = 3, *p* < 0.011), reaching statistical significance at 5 μg/L (*p* = 0.009) and 45 μg/L (*p* = 0.002) (Figure 1c). In addition, SVL was significantly increased in all TCC-treated groups relative to the control (*H* = 18.354, df = 3, *p* < 0.001) (Figure 1d). These morphological changes may further affect the normal physiological processes in tadpoles.

**Table 1:** Acute toxicity of the TCC to *R. taihangensis* tadpoles.

Duration of exposure (h)	PROBIT model^a^	Lethal concentration 50% (LC_50_) (μg/L)	95% confidence intervals
24	PROBIT(*p*) = −12.301 + 4.850*X*	343.708	292.996–399.527
48	PROBIT(*p*) = −8.676 + 3.679*X*	228.342	120.658–308.417
72	PROBIT(*p*) = −9.866 + 4.298*X*	197.477	84.442–273.851
96	PROBIT(*p*) = −12.037 + 5.398*X*	169.863	98.799–223.109

a PROBIT model: PROBIT(*p*) = intercept + B*X* (covariates *X* are transformed using the base 10 logarithm).

### 3.2 Influence of TCC exposure on the gut microbiota

**Figure 2:**
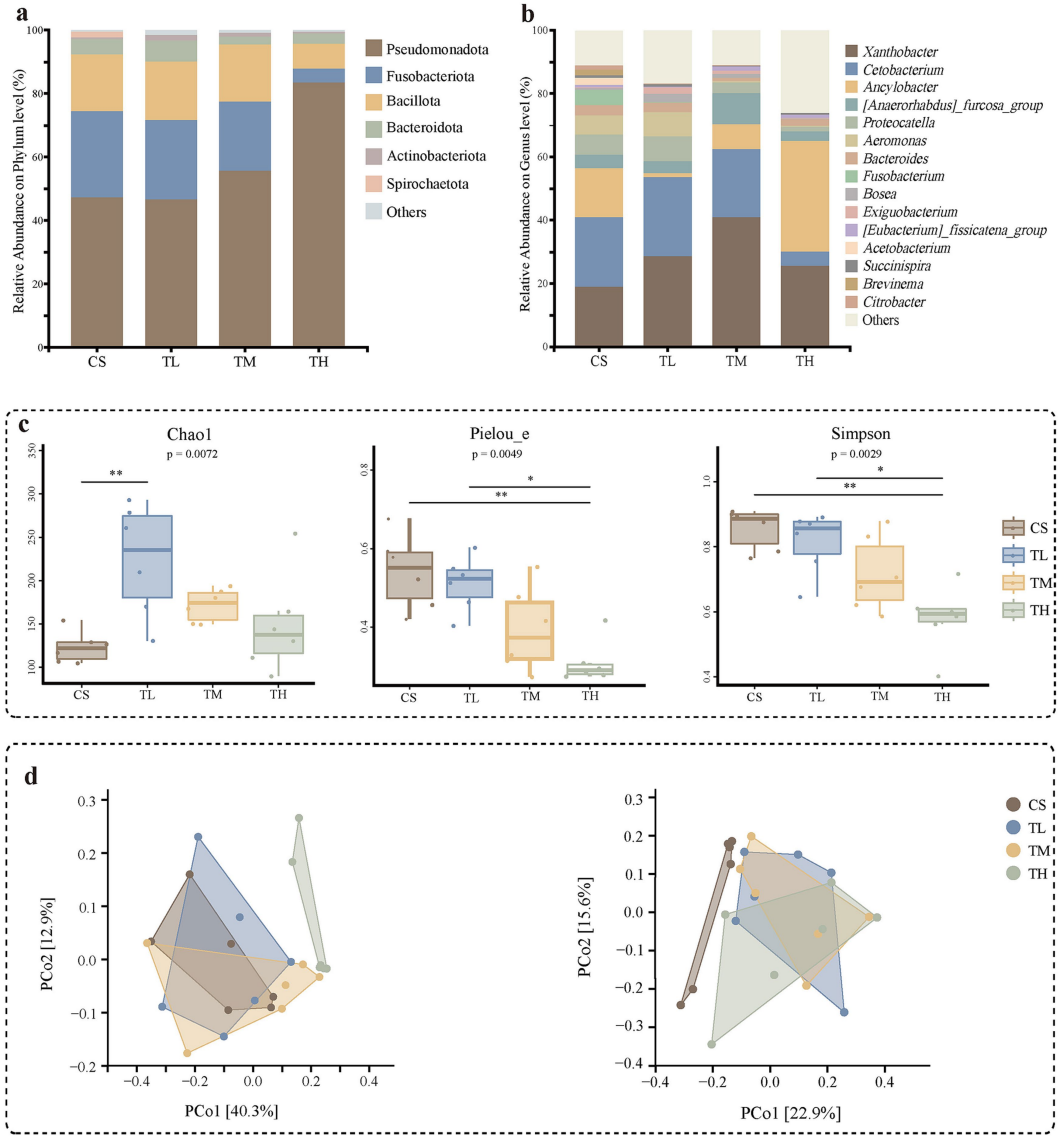
Gut microbiota of *R. taihangensis* tadpoles exposed to solvent control (CS), 5 μg/L (TL), 15 μg/L (TM), and 45 μg/L (TH) of TCC for 3 weeks. The present study used a bar graph to examine the microbiota existing at the phylum **(a)** and genus **(b)** levels. The presented figure exclusively displays phyla and genera that demonstrate relative abundances surpassing 1% in at least one of the samples. The comparison was based on the Chao1, Pielou’s evenness and Simpson indices **(c)**. Statistical analysis was performed using Kruskal–Wallis test and Dunn’s *post hoc* test. Significance levels were indicated as *and **for 0.01 < *p* < 0.05, 0.001 < *p* < 0.01, respectively. PCoA scatter plots presenting the variations in beta-diversity were based on the weighted UniFrac and unweighted UniFrac distances **(d)**. Adonis test with 999 permutations was also calculated to assess the difference in the bacterial diversity among treatments.

In the gut microbiota of *R. taihangensis* tadpoles, the dominant phyla across all groups (i.e., the three TCC treatments and the solvent control) were Pseudomonadota (Proteobacteria), Fusobacteriota, Bacillota (Firmicutes) and Bacteroidota (Figure 2a). At the genus level, the most abundant taxa were *Xanthobacter*, *Cetobacterium*, *Ancylobacter*, *[Anaerorhabdus]_furcosa_group*, *Proteocatella* and *Aeromonas* (Figure 2b). Compared to the solvent control, Kruskal–Wallis tests showed that low and medium TCC concentrations did not induce significant changes in the phylum-level abundance. However, the high-concentration TCC group exhibited a significant increase in Pseudomonadota and a significant decrease in Fusobacteriota (Supplementary Figure S2a), suggesting that Pseudomonadota and Fusobacteriota were more sensitive to TCC, whereas Bacillota and Bacteroidota appeared relatively insensitive. At the genus level, *Cetobacterium* and *Citrobacter* showed notable sensitivity to TCC. *Cetobacterium* abundance decreased significantly only under high TCC exposure, whereas *Citrobacter* was significantly reduced in all TCC-treated groups (Supplementary Figure S2b).

Alpha-diversity of the gut microbiota was evaluated using the Chao1, Simpson and Pielou’s evenness indices (Figure 2c). The Chao1 index, reflecting ASV richness, increased significantly (*p* < 0.01; Figure 2c) only in the low-concentration TCC group (TL, 5 μg/L). Overall, species richness was lowest in the solvent control group (CS) and highest in the TL group, declining gradually as TCC concentrations increased from TL to medium (TM, 15 μg/L) and high (TH, 45 μg/L) levels. Nevertheless, median richness in all three TCC-exposed groups remained above that of the CS group (Figure 2c). Pielou’s evenness index decreased progressively from CS to TL, TM, and TH (*p* = 0.0049), indicating a gradual reduction in community evenness relative to the control (CS) (Figure 2c). Similarly, the Simpson index, which reflects species dominance and distribution uniformity, also decreased continuously across the same gradient (*p* = 0.0029), with a statistically significant reduction observed only in the TH group (Figure 2c). Together, these indices revealed a shift in the gut microbiota structure: species richness initially increased then decreased, while dominance increased and evenness declined, reflecting a transition from a “basically balanced” state to “rich but imbalanced,” and finally to a “depleted and imbalanced” microbial community. Beta-diversity, evaluated by PCoA based on both weighted UniFrac and unweighted UniFrac distances, indicated that the high-concentration TCC group (TH) diverged significantly from the control in both metrics (*p* < 0.05, Figure 2d). Unweighted UniFrac analysis revealed significant structural shifts even in the TL and TM groups compared to the CS group (*p* < 0.05, Figure 2d).

**Figure 3:**
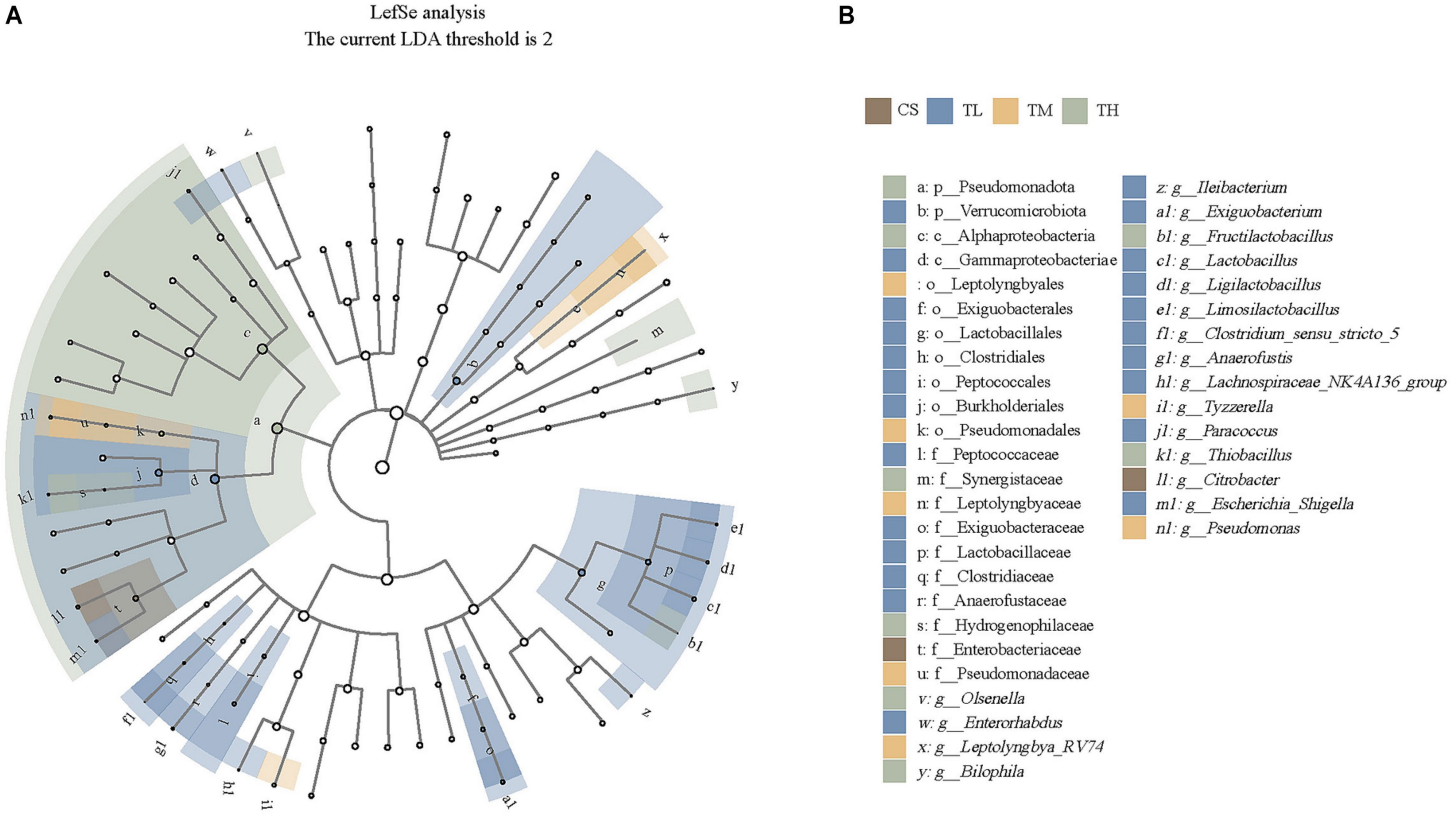
Differentially abundant intestinal taxa occurring in *R. taihangensis* tadpoles following exposure to solvent control (CS), 5 μg/L (TL), 15 μg/L (TM), and 45 μg/L (TH) of TCC. **(a)** Cladogram generated from LEfSe analysis of differentially abundant taxa with *p* < 0.05 and LDA score >2 in solvent control and TCC groups. Rings from inside to outside represent the taxonomic levels from phylum to genus. **(b)** LDA scores of differentially abundant intestinal taxa in solvent control and TCC groups (LDA score >2). p, phylum; c, class; o, order; f, family; g, genus. Different groups and the corresponding differential bacteria are marked in different colors.

LEfSe analysis was performed across multiple taxonomic levels (from phylum to genus) to identify specific bacterial taxa associated with TCC exposure. At the phylum level, Verrucomicrobiota and Pseudomonadota were microbial markers in the 5 μg/L and 45 μg/L TCC groups, respectively (Figure 3). At the genus level, *Citrobacter* was identified as a marker genus in the control group (Figure 3). In TCC-exposed tadpoles, the 5 μg/L group was characterized by enrichment of *Exiguobacterium*, *Anaerofustis* and *Paracoccus*; the marker microbial genera in 15 μg/L group were *Tyzzerella*, *Pseudomonas* and *Leptolyngbya_RV74*; while in the 45 μg/L group, the marker microbial genera were *Citrobacter*, *Bilophila* and *Thiobacillus* (Figure 3). Notably, the 5 μg/L of TCC exposure yielded a more distinct set of bacterial markers compared to the other exposure concentrations (Figure 3). In total, 19 microbial markers were identified at the genus level.

### 3.3 Influence of TCC exposure on metabolome

**Figure 4:**
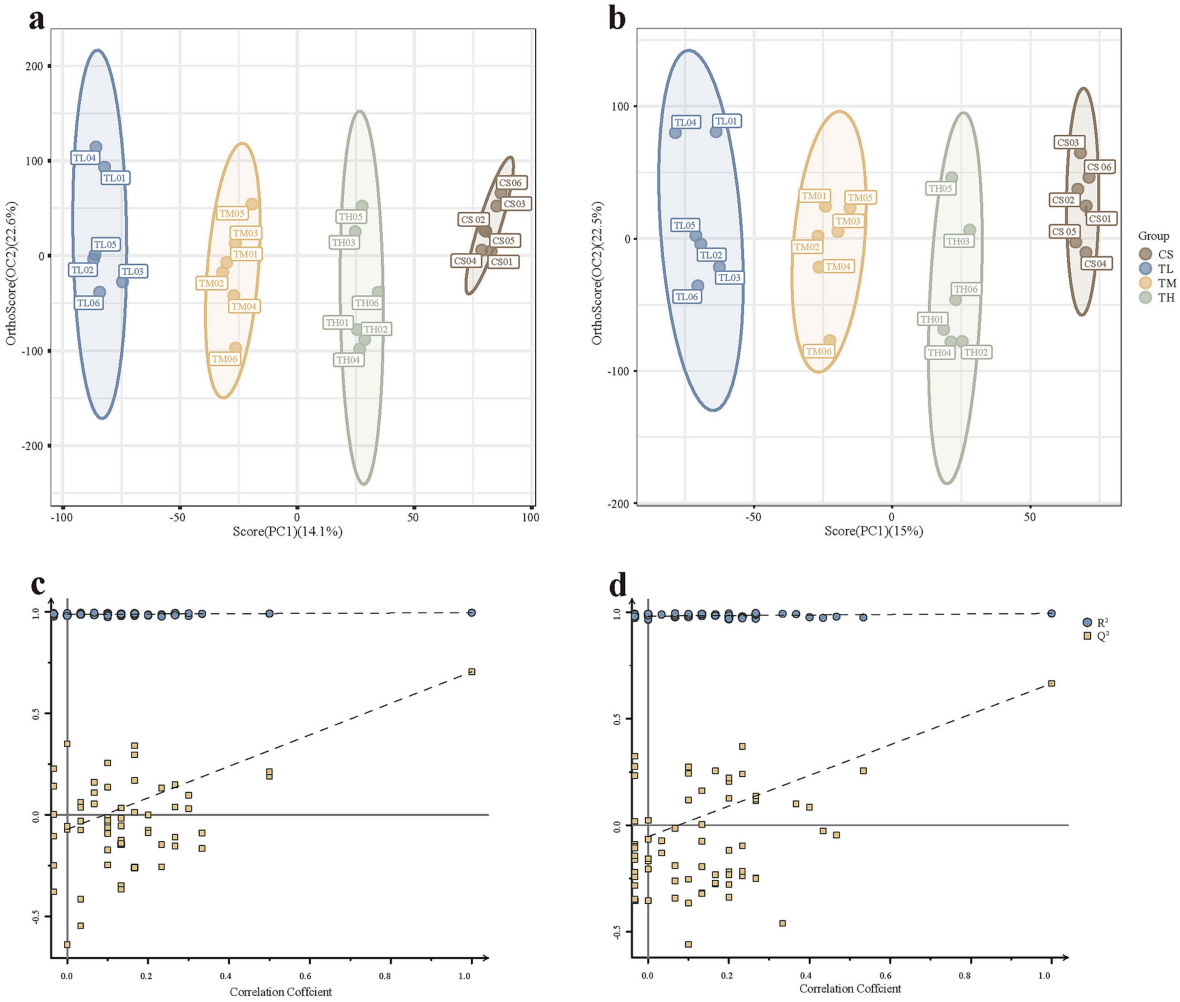
The non-targeted metabolomics profiling analysis of tadpoles. The OPLS-DA plots of CS (solvent control), TL (5 μg/L), TM (15 μg/L), and TH (45 μg/L) groups in positive **(a)** and negative **(b)** ion modes. *R*^2^*Y* represent the explained variance of the constructed models for the *Y* matrices, while *Q*^2^ indicates model predictive capability. Validation plots were obtained in **(c)** positive mode (*R*^2^ = 0.996, *Q*^2^ = 0.705) and **(d)** negative mode (*R*^2^ = 0.995, *Q*^2^ = 0.666).

Non-targeted metabolomic profiling detected a total of 28,202 and 18,488 features in positive and negative ionization modes, respectively. OPLS-DA revealed a clear and distinct separation among all groups in both positive and negative modes (Figures 4a,b). Model quality had high robustness, as indicated by *R*^2^*Y* and *Q*^2^ values close to 1 in positive (*R*^2^*Y* = 0.996, *Q*^2^ = 0.705) and negative (*R*^2^*Y* = 0.995, *Q*^2^ = 0.666) ion modes. Permutation tests further confirmed model validity and absence of overfitting (Figures 4c,d).

**Figure 5:**
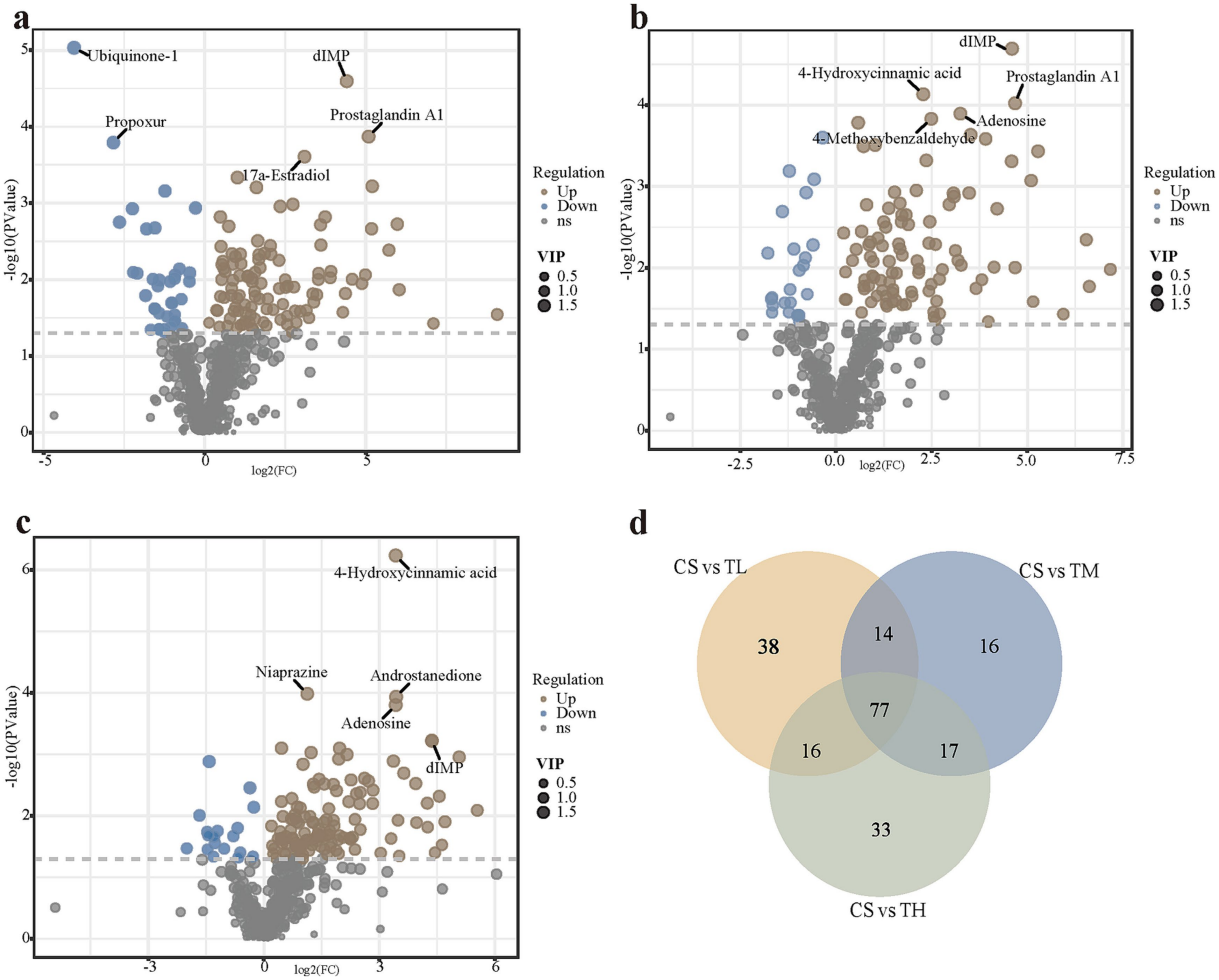
Differential metabolites. Volcano plot **(a–c)**: the size of the point represents the VIP value. The brown dot represented the up-regulated differences, the blue dot represented the down-regulated differences, and the gray dot represented the metabolites that did not meet the differential screening conditions. **(a)** CS vs. TL. **(b)** CS vs. TM. **(c)** CS vs. TH. Venn diagram **(d)** showed the common differential metabolites of CS vs. TL, CS vs. TM, and CS vs. TH. CS, solvent control group; TL, 5 μg/L group; TM, 15 μg/L group; TH, 45 μg/L group.

**Figure 6:**
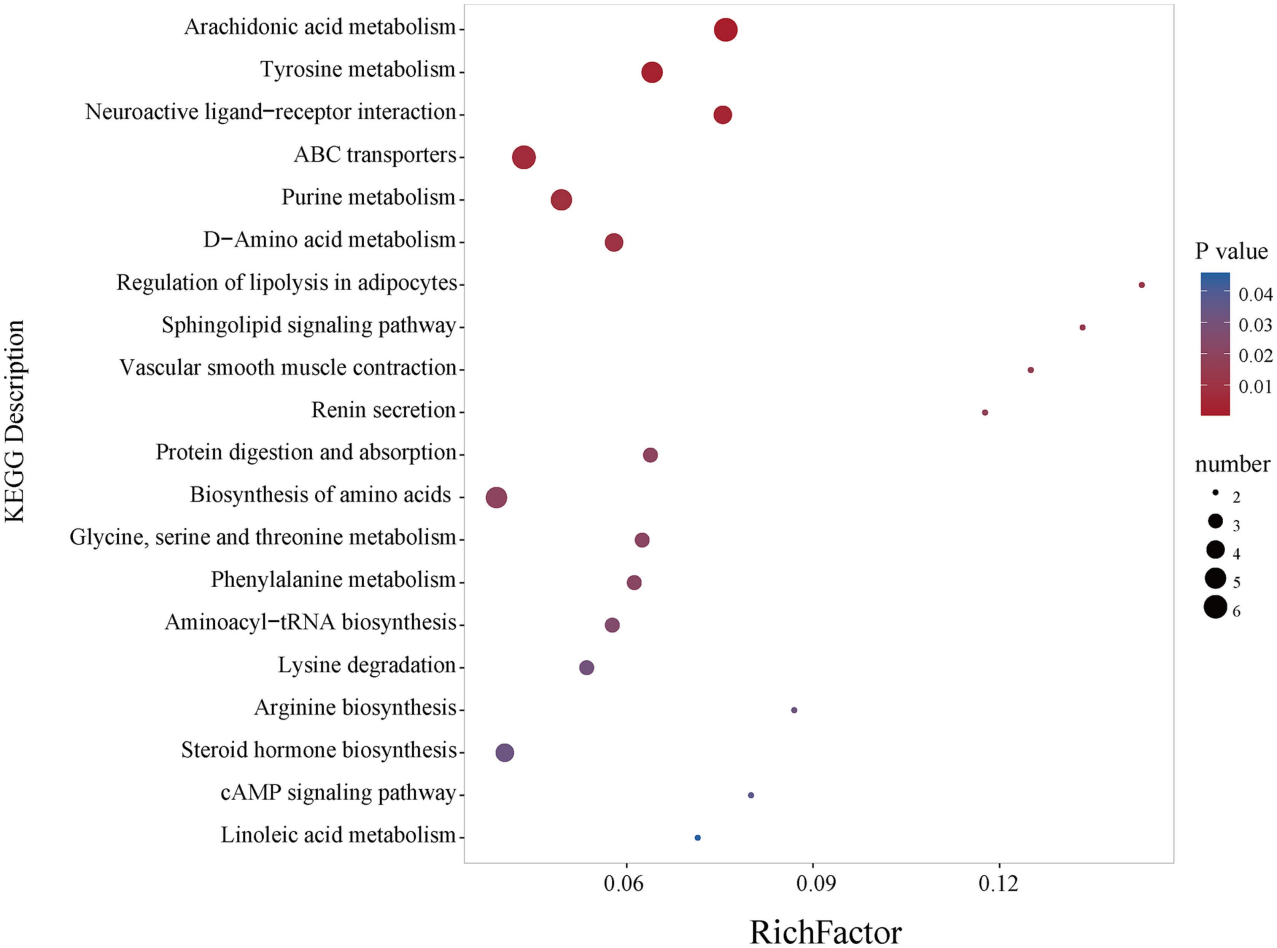
Pathway analysis of the overlapped metabolites of low, medium, and high exposure.

Using the criteria of VIP >1 and *p* < 0.05, 145 differentially expressed metabolites were identified in the low-concentration TCC group (37 down-regulated, 108 up-regulated), 124 in the medium-concentration group (24 down-regulated, 100 up-regulated), and 143 in the high-concentration group (18 down-regulated, 125 up-regulated) (Figures 5a–c). A Venn diagram showed that 77 metabolites were common to all three comparison groups (Figure 5d and Supplementary Table S2). These differential metabolites were primarily classified at the HMDB superclass level as lipids and lipid-like molecules, organic acids and derivatives, benzenoids and organoheterocyclic compounds (Supplementary Table S2). KEGG enrichment analysis of the 77 shared metabolites identified 21 significantly altered metabolic pathways. The top 20 pathways, ranked by significance, were presented in a bubble chart (Figure 6 and Supplementary Table S3). Among these, five pathways (Arachidonic acid metabolism, tyrosine metabolism, ABC transporters, purine metabolism, and biosynthesis of amino acids) were selected based on a *p*-value <0.05 and the inclusion of at least five differential metabolites. A total of 23 metabolites enriched in these pathways were sorted out (Supplementary Table S4).

### 3.4 Integrated analysis of microbiota and metabolome

An integrated multi-omics approach was performed to investigate the relationship between gut microbiota and metabolic alterations following TCC exposure. Across the three comparison groups, 210 differential metabolites were identified (Figure 5d). LEfSe analysis further detected 86 significant metabolic markers across all experimental groups (Supplementary Table S5). Spearman correlation analysis revealed 15 strongly correlated microbe-metabolite pairs (|rho| >0.7), comprising 12 positive and three negative correlations (Supplementary Table S6). Differences in the metabolic markers across groups are presented in Supplementary Figure S3.

## 4 Discussion

In the present study, we employed an integrated multi-omics strategy, combining morphological assessment, gut microbiome analysis, and untargeted metabolomics, to systematically evaluate the toxicological effects of TCC on *R. taihangensis* tadpoles at environmentally relevant concentrations (5, 15, and 45 μg/L). Our results demonstrated that TCC exposure initiated a series of adverse effects across multiple biological levels: it induced phenotypic changes (e.g., reduced body condition score), disrupted gut microbial homeostasis (manifested as dysbiosis), and further disturbed essential metabolic pathways (e.g., arachidonic acid metabolism and purine metabolism). To our knowledge, this is the first study to integrate morphological, microbial, and metabolic data in *R. taihangensis* tadpoles, thereby unraveling the sublethal toxicity mechanisms of TCC. These findings address important knowledge gaps on TCC-induced ecotoxicity in amphibians, a group irreplaceable to aquatic ecosystem integrity.

### 4.1 Potential morphological impacts of TCC exposure

Although chronic TCC exposure did not significantly affect body mass or total length of *R. taihangensis* tadpoles, it induced two conspicuous phenotypic changes: an increase in SVL and a reduction in body condition score. The significant increase in SVL, without a corresponding change in total length, suggested that TCC may disrupt developmental allometry. Such a premature shift in body proportions could desynchronize morphological development from critical environmental cues (e.g., food availability), potentially resulting in asynchronous metamorphosis—a known factor that reduces post-metamorphic survival (Székely et al., 2020). This risk is particularly critical for *R. taihangensis*, a species whose mountain-stream habitat already imposes strict constraints on metamorphic timing due to factors such as short growing seasons.

The observed reduction in body condition score, calculated as 100 × (body mass/body length^3^), indicated depleted somatic energy reserves (Ruthsatz et al., 2020). This energy deficit undermines the tadpoles’ capacity to cope with environmental stresses, including food deprivation, pathogen infection, and environmental pollution (Ruthsatz et al., 2020). For *R. taihangensis*, which inhabits food-limited mountain streams, this TCC-induced energy deficit could lead to heightened population vulnerability through reduced overwintering survival or metamorphic success. Furthermore, the non-significant trend of increased total length without a corresponding increase in body mass suggested that TCC might disrupt energy allocation, favoring linear growth over mass accumulation. This trade-off could compromise long-term fitness, given that body mass is a key determinant of post-metamorphic reproductive success in amphibians (Tejedo, 1992). Collectively, these morphological changes demonstrated that TCC disrupts the normal developmental trajectory of *R. taihangensis* tadpoles, with potential implications for individual survival and population persistence.

### 4.2 TCC-induced shifts in gut microbiota composition and potential function

The intestinal microbiota plays a critical role in host nutrient absorption, digestion and immune function (Dawood and Koshio, 2016; Gao et al., 2020). Numerous studies have shown that environmental pollutants can alter gut microbiota diversity and composition (Lv et al., 2023; Zhang Q. et al., 2023; Zhu et al., 2023). In this study, Pseudomonadota, Fusobacteriota, Bacillota and Bacteroidota collectively constituted approximately 97% of the intestinal bacterial communities in *R. taihangensis* tadpoles. We found that TCC exposure significantly reduced gut microbial diversity and disrupted intestinal homeostasis, which aligns with previously reported effects in *R. chensinensis* (Yang et al., 2020b) and mice (Song et al., 2022; Zhang et al., 2021).

#### 4.2.1 Changes in microbial diversity

Alpha-diversity analysis revealed a concentration-dependent shift in gut microbiota structure: the Chao1 index (ASV richness) increased significantly in the low-concentration group (5 μg/L) but declined with increasing TCC concentrations, while both Pielou’s evenness index and Simpson index decreased progressively across all TCC groups. This pattern reflects a transition from a “basically balanced” microbial community (control group) to a “rich but imbalanced” state (low TCC) and finally to a “depleted and imbalanced” state (high TCC). Decreased intestinal microbial diversity is increasingly recognized as a risk factor for various animal diseases (Marchesi et al., 2016; Serra et al., 2018), as it may compromise essential microbial functions (e.g., vitamin synthesis, short-chain fatty acid production). Thus, the TCC-induced decline in microbial diversity likely contributes to the adverse health effects observed in *R. taihangensis* tadpoles.

Beta-diversity analysis (PCoA) further confirmed that TCC disrupts gut microbial structure: the high-concentration group (45 μg/L) diverged significantly from the control in both weighted and unweighted UniFrac distances, while even low (5 μg/L) and medium (15 μg/L) concentrations induced significant shifts in unweighted UniFrac distances. Weighted UniFrac emphasizes the abundance of dominant taxa, whereas unweighted UniFrac focuses on rare taxa (Lozupone et al., 2007). The significant shift in unweighted UniFrac at low TCC concentrations suggests that rare bacterial taxa, often involved in specialized functions (e.g., pollutant degradation), are particularly sensitive to TCC. This sensitivity may further impair microbial community resilience, as rare taxa contribute to functional redundancy and ecosystem stability (Yang et al., 2024).

#### 4.2.2 Changes in key microbial taxa

Taxonomic analysis identified specific bacterial taxa that are sensitive to TCC, with perturbations in community composition observed at both the phylum and genus levels. Furthermore, comparative taxonomic profiling revealed a significant increase in the relative abundance of the phylum Pseudomonadota in the high-concentration TCC group (45 μg/L) compared to the solvent control group. Pseudomonadota is a dominant phylum in many amphibian gut microbiomes (Chen Z. et al., 2022), but elevated abundance of this phylum is widely recognized as a marker of microbial dysbiosis and disease risk (Shin et al., 2015). For example, increased levels of Pseudomonadota have been associated with the IBD, chronic kidney disease (CKD) and neurodegenerative Alzheimer’s disease, potentially through mechanisms involving intestinal barrier disruption, oxidative stress and chronic inflammation (Morgan et al., 2012; Andersen et al., 2017; Vogt et al., 2017). An increase in Pseudomonadota has also been observed in *Bufo gargarizans* following Pb exposure (Chai et al., 2023), further supporting its role as a responsive taxon in amphibian gut microbiota under pollutant stress. In contrast, the abundance of Fusobacteriota was significantly reduced in TCC-exposed tadpoles. A decline in Fusobacterium induced by environmental pollutants may impair normal immune system development and weaken the body’s defense against bacterial infections (Knutie et al., 2017). Additionally, reduced levels of Fusobacterium have been linked to intestinal tissue necrosis (Knutie et al., 2017; Wan et al., 2022). Consistently, a decrease in Fusobacterium abundance caused by heavy metal exposure has been shown to contribute to metabolic disorders and infectious diseases in *B. gargarizans* (Zheng et al., 2020), supporting our hypothesis that TCC-induced Fusobacteriota depletion compromises tadpole health.

Our results also demonstrated that high-concentration TCC exposure significantly reduced the abundance of the genus *Cetobacterium* compared to the solvent control group. *Cetobacterium* is known to contribute to the fermentation of peptides and carbohydrates (Wang A. et al., 2021) and participates in vitamin B_12_synthesis (Sugita et al., 1991). In particular, *Cetobacterium somerae* has been shown to synthesize vitamin B_12_, which can modulate the intestinal redox state, influence the composition and function of the gut microbiome, and enhance interactions between the microbiota and intestinal tight junctions, thereby improving host resistance to pathogen infection (Qi et al., 2023). In zebrafish, *Cetobacterium* has been implicated in glucose homeostasis regulation via parasympathetic nerve activation, suggesting its role in host carbohydrate metabolism (Wang A. et al., 2021). A decline in *Cetobacterium* may also suppress immune-related genes expression in tadpole liver (Wang Z. Y. et al., 2024). Consistently, a significant reduction in *Cetobacterium* was observed in *R. zhenhaiensis* after Pb exposure, indicating heightened risk of dysbiosis and health impairment (Li et al., 2024). Additionally, that the abundance of the genus *Citrobacter* was significantly lower in all three TCC-treated groups relative to the solvent control group. As intestinal microbial community play important roles in tadpole growth and development (Chai et al., 2023), a reduction in *Citrobacter* has previously been associated with delayed or abnormal development in tadpoles (Yang et al., 2020a). The morphological changes observed in TCC-exposed tadpoles in this study further supported the notion that decreased *Citrobacter* may adversely affect growth and development. *Citrobacter* is considered a key constituent of the gut microbiota, potentially involved in immune system training and regulation. Its depletion may affect the host’s immune defense against other intestinal pathogens (Collins et al., 2014).

LEfSe analysis identified additional taxonomic markers of TCC exposure: the 5 μg/L group was enriched in *Exiguobacterium* and *Paracoccus* (potential pollutant degraders), the 15 μg/L group in *Pseudomonas* (known for antibiotic resistance), and the 45 μg/L group in *Bilophila* (linked to intestinal inflammation). These markers highlight concentration-specific microbial responses to TCC, suggesting that low TCC concentrations may select for pollutant-tolerant taxa, while high concentrations favor pro-inflammatory taxa. It is important to note that gut microbial communities form a highly interconnected ecosystem. Alterations in one bacterial group can trigger cascading effects on other symbiotic or competing species, ultimately disrupting microbial balance and impairing the structural and functional integrity of the intestinal microbiota (Wang Y. et al., 2024).

### 4.3 TCC-induced metabolic perturbations and potential hazard

Non-targeted metabolomics revealed that TCC exposure disrupts the metabolome of *R. taihangensis* tadpoles, with 145, 124, and 143 differential metabolites identified in the low-, medium-, and high-concentration groups, respectively. Among these, 77 metabolites were shared across all TCC groups, primarily classified as lipids/lipid-like molecules, organic acids, and benzenoids. KEGG enrichment analysis of these shared metabolites identified five key perturbed pathways: arachidonic acid metabolism, tyrosine metabolism, ABC transporters, purine metabolism, and biosynthesis of amino acids. These pathways are critical for immune function, neuroendocrine signaling, energy homeostasis, and nutrient utilization—providing molecular insights into TCC’s sublethal toxicity.

#### 4.3.1 Disturbance of the arachidonic acid metabolism pathway

Arachidonic acid metabolites are essential for immune surveillance, inflammation resolution, neuronal signaling, and glucose metabolism (Hanna and Hafez, 2018). Our results showed that six metabolites (i.e., 20-carboxy-leukotriene B_4_, 11b-PGF2a, hepoxilin B_3_, 14,15-DiHETrE, 16(R)-HETE and leukotriene E_4_) associated with the arachidonic acid metabolism pathway were significantly up-regulated in the TCC-exposed groups. Among these, 20-carboxy-leukotriene B_4_ and PGF2a are known to regulate inflammation, immune responses and vasoconstriction (Seeger et al., 1991; Wanderer et al., 2018; Wang B. et al., 2021). Hepoxilins, detected at elevated levels in psoriatic lesions, have been implicated in the pathogenesis of inflammatory skin diseases (Antón et al., 2002). Additionally, 14,15-DiHETrE serves as a stable hydrolysis product of anti-inflammatory 14,15-EETs, mediated by soluble epoxide hydrolase (sEH) and can indirectly reflect 14,15-EET levels (Yang et al., 2013). Given that TCC has been identified as a potent *in vitro* inhibitor of sEH, it might affect the levels of 14,15-DiHETrE through this mechanism (Liu et al., 2011). 16(R)-HETE contributes to the regulation of vasodilation and vasoconstriction, inflammatory processes, and cell proliferation (Bednar et al., 2000), while leukotriene E_4_ is an inflammatory mediator derived from arachidonic acid via the 5-LOX pathway (Wang and Dubois, 2010). Under physiological conditions, arachidonic acid metabolites facilitate inflammatory cell chemotaxis and enhance vascular permeability during inflammatory responses. The up-regulation of these metabolites in TCC-exposed tadpoles suggested enhanced immune cell recruitment and activation, as well as an amplified local inflammatory reaction, which may ultimately affect the host’s ability to eliminate pathogens or repair tissue damage.

#### 4.3.2 Disturbance of the tyrosine metabolism pathway

The tyrosine metabolic pathway has been shown to play significant roles in the neurotransmitter synthesis, mood regulation, thyroid hormone synthesis, immune function (Dimić et al., 2018; Ko et al., 2020). In the present study, four up-regulated metabolites (i.e., 4-hydroxycinnamic acid, norepinephrine, 3-hydroxyphenylacetic acid and vanillylmandelic acid) and one down-regulated (3-methoxy-4-hydroxyphenylglycolaldehyde) metabolite were identified within this pathway. 4-hydroxycinnamic acid has been reported to suppress ovalbumin-induced airway inflammation in allergic asthma (Ko et al., 2020). Norepinephrine, an important neurotransmitter, influences stress response and emotional regulation, and alterations in its metabolic profile may reflect physiological adaptations to environmental stressors (Dimić et al., 2018). 3-hydroxyphenylacetic acid, a flavonoid-derived metabolite, modulates intestinal microbial activity and thus may affect host health (Dias et al., 2022). Vanillylmandelic acid is a major end metabolite of adrenaline and norepinephrine, and it can serve as a clinical marker in tumor screening (Dimić et al., 2018). Furthermore, 3-methoxy-4-hydroxyphenylglycolaldehyde is regarded as a reliable indicator of central norepinephrine turnover (Elsworth et al., 1983). The observed up-regulation of 4-hydroxycinnamic acid, norepinephrine, 3-hydroxyphenylacetic acid, and vanillylmandelic acid suggested elevated neural activity or enhanced stress responses following TCC exposure. Conversely, the down-regulation of 3-methoxy-4-hydroxyphenylglycolaldehyde might reflect disrupted norepinephrine metabolism. Based on the functional relevance of these altered metabolites, we proposed that TCC exposure perturbs nervous system function, endocrine signaling, and stress adaptation of *R. taihangensis* tadpoles.

#### 4.3.3 Disturbance of the ABC transporters pathway

ABC transporters are a class of widely distributed membrane proteins that utilize ATP hydrolysis to drive the transport of various substances (i.e., ions, metabolites, drugs and toxins) across biological membranes (Rees et al., 2009). Our results revealed significant alterations in several metabolites associated with the ABC transporters pathway following TCC exposure. Specifically, betaine, L-lysine, L-phenylalanine, L-aspartic acid and adenosine were significantly up-regulated in all the three TCC-treated groups. In contrast, nopaline was significantly down-regulated in the low-concentration group but up-regulated in the medium- and high-concentration groups. Betaine has been shown to support liver function and exert hepatoprotective, anti-inflammatory, and antioxidant effects (Arumugam et al., 2021). Lysine, an essential amino acid, is indispensable for animal growth and dietary nutrition (Matthews, 2020). L-phenylalanine, another essential amino acid, is a precursor for the synthesis of adrenaline, thyroxine and melanin; its up-regulation might influence the synthesis of these important hormones and pigments, thereby affecting physiological function (Slominski et al., 2012). L-aspartic acid plays diverse roles in health and disease, contributing to protein and nucleotide synthesis, gluconeogenesis, urea and purine-nucleotide cycles, and neurotransmission (Holeček, 2023). Adenosine acts as a key intermediate metabolite involved in processes such as lipolysis (Johansson et al., 2007), immune function (Rosenberger et al., 2009), inflammatory (Haskó et al., 2008; Eltzschig and Carmeliet, 2011) and neurodegenerative disorders (Fredholm, 2007). Nopaline, a known plant metabolite (Zanker et al., 1992), exhibited a concentration-dependent shift in abundance under TCC exposure. Collectively, these metabolite changes reflect complex regulatory adjustments in metabolic pathways in response to environmental stress. The up-regulation of amino acids and related metabolites (i.e., betaine, L-lysine, L-phenylalanine, L-aspartic acid and adenosine) might suggest cellular adaptation to stressors such as hypertonic conditions, pathogen invasion, or nutritional deficiency. These changes could represent compensatory mechanisms under adverse conditions (e.g., malnutrition, stress, pathological state, etc.) and may also signal dysregulation in specific metabolic pathways or the onset of disease.

#### 4.3.4 Disturbance of the purine metabolism pathway

In addition to producing the structural units of DNA and RNA, purine metabolism plays essential roles in cellular energy supply, intracellular signaling, and the regulation of cell survival and proliferation (Pedley and Benkovic, 2017). Our metabolomics analysis revealed significant up-regulation of several metabolites involved in purine metabolism, including hypoxanthine, dIMP, guanine, xanthylic acid and adenosine. Hypoxanthine contributes to intestinal epithelial energy metabolism and helps maintain intestinal barrier integrity (Lee et al., 2018). Both hypoxanthine and guanine are key intermediates in the purine metabolic pathway, where they are converted to inosine 5′-mono-phosphate (IMP) and guanosine 5′-monophosphate (GMP) by hypoxanthine-guanine phosphoribosyltransferase (HPRT), a critical step in the purine recycling and nucleotide synthesis (Pedley and Benkovic, 2017). Up-regulation of hypoxanthine and guanine may thus influence intracellular IMP and GMP levels, subsequently affecting ATP synthesis and overall cell energy homeostasis. Xanthylic acid acts as a key intermediate in purine nucleotide metabolism and participates in GMP synthesis (Ballut et al., 2023). Adenosine exerts immunomodulatory functions by binding to G protein-coupled surface receptors (Haskó et al., 2004; Haskó et al., 2008) and has been reported to confer neuroprotective effects against ischemic brain injury via adenosine receptor activation (Seydyousefi et al., 2019). Additionally, 2′-deoxy-hypoxanthine-5′-monophosphate (dIMP), formed by the deamination of dAMP in DNA, exhibits mutagenic potential (Saparbaev et al., 2000). In summary, the coordinated up-regulation of hypoxanthine, dIMP, guanine, xanthylic acid and adenosine suggested an overall activation of the purine metabolic pathway in response to TCC exposure. Such changes often reflect cellular adaptation to environmental challenges, including hypoxia, cellular damage, enhanced proliferation signaling, or immune activation.

#### 4.3.5 Disturbance of the biosynthesis of amino acids pathway

In addition to serving as the fundamental structural units of proteins, amino acids act as crucial precursors for numerous metabolites, which are essential for many biological processes, including cell growth, division and metabolic signaling pathways (Idrees et al., 2020). Our metabolomics analysis revealed significant up-regulation of several metabolites (i.e., L-lysine, L-phenylalanine, citrulline, L-aspartic acid and L-2,4-diaminobutyric acid) involved in amino acid biosynthesis. As previously discussed in the context of ABC transporters, L-lysine, L-phenylalanine, and L-aspartate contributed importantly to protein synthesis, nerve conduction, and hormone production. Citrulline, a non-essential amino acid predominantly synthesized by intestinal mucosal cells, has been identified as a biomarker for evaluating functional intestinal epithelial mass and identifying intestinal failure (Crenn et al., 1998; Bahri et al., 2013). Meanwhile, L-2,4-diaminobutyric acid is a neurotoxic non-protein amino acid produced by *Cyanobacteria* and it has been associated with neurodegenerative disorders in animals and humans (Spasic et al., 2018). The coordinated up-regulation of these amino acids likely reflects metabolic adaptation to physiological or pathological challenges. Under environmental stress or metabolic disorders, organisms appear to modulate amino acid biosynthesis to optimize resource allocation and maintain metabolic equilibrium, thereby supporting the homeostatic maintenance of essential physiological functions.

### 4.4 Integrated analysis of microbiota and metabolome

To validate the link between gut microbiota and metabolic perturbations, we performed Spearman correlation analysis on LEfSe-identified microbial and metabolic markers (LDA score >2.0). This analysis revealed 15 strongly correlated microbe-metabolite pairs (|rho| >0.7), confirming that TCC-induced gut dysbiosis contributes to metabolic dysfunction.

*Lactobacillus* contributes to host health and microbial balance through multiple mechanisms, including immune homeostasis regulation (Rizzello et al., 2011), intestinal barrier maintenance (Zhao et al., 2021), metabolic function modulation (Teng et al., 2020) and pathogens inhibition (Alakomi et al., 2000). Our results revealed a significant negative correlation between *Lactobacillus* and Isovitexin (rho = −0.76, *p* < 0.001). This relationship is supported by recent studies demonstrating bidirectional interaction between the two: *Lactobacillus* can enhance the bioavailability of isovitexin by secreting β-glucosidases that hydrolyze its glycosidic form into more absorbable aglycones, while isovitexin selectively promotes the growth of *Lactobacillus*, thereby reinforcing its antioxidant and anti-inflammatory effects (Baky et al., 2022). In addition, *Lactobacillus reuteri* Zj617 has been shown to serve as a substrate source for microbial spermidine biosynthesis, counteracting the loss of spermidine-producing taxa and protecting against metabolic disorders (Ma et al., 2025). At the same time, we also found a significant positive correlation between *Lactobacillus* and Prostaglandin A1 (PGA1) (rho = 0.72, *p* < 0.001). In line with this, Zhao et al. (2022) reported that both *Lactobacillus* and PGA1 were positively associated with pro-inflammatory cytokines (e.g., TNF-*α*, IL-6, CXCL1) in brain tissues, suggesting that *Lactobacillus* may promote sepsis-related neuroinflammation via PGA1 up-regulation (Zhao et al., 2022). Conversely, a recent study by Shen et al. (2024) demonstrated that oral administration of *Lactobacillus plantarum* L168 increased PGA1 levels in a rat model of bronchopulmonary dysplasia (BPD), where both were implicated in anti-inflammatory and lung injury repair processes (Shen et al., 2024). Furthermore, *Limosilactobacillus* has been shown to protect intestinal epithelial integrity, alleviate inflammation (Xu et al., 2025), modulate oxidative responses (Choi et al., 2024), and regulate immune function (Abramov et al., 2022). A recent study reported that L-asparagine (L-ASNase, E.C.3.5.1.1) produced by *Limosilactobacillus secaliphilus* catalyzes the hydrolysis of L-asparagine to L-Aspartic acid and NH_3_ (Zhang et al., 2024), consistent with our findings of strong positive correlation between *Limosilactobacillus* and L-aspartic acid (rho = 0.72, *p* < 0.001).

Integrating our findings across morphology, microbiome, and metabolome, we propose a causal chain explaining TCC’s sublethal toxicity to *R. taihangensis* tadpoles. (1) TCC exposure disrupts gut microbiota: TCC preferentially inhibits the growth of beneficial taxa (e.g., *Cetobacterium*, *Citrobacter*) and promotes the abundance of pro-inflammatory taxa (e.g., Pseudomonadota). This leads to reduced microbial diversity, impaired intestinal barrier integrity, and loss of key microbial functions (e.g., vitamin B_12_ synthesis). (2) Microbial dysbiosis drives metabolic perturbations: The depletion of *Cetobacterium* reduces vitamin B_12_ availability, impairing purine metabolism and leading to the accumulation of purine intermediates (e.g., hypoxanthine) and reduced ATP production. Concurrently, the decline in *Citrobacter* and other beneficial taxa disrupts amino acid biosynthesis and ABC transporter function, leading to perturbations in tyrosine metabolism (e.g., norepinephrine up-regulation) and arachidonic acid metabolism (e.g., pro-inflammatory metabolite up-regulation). (3) Metabolic perturbations induce phenotypic changes: Reduced ATP production leads to a decline in body condition score, as energy is diverted from somatic growth to stress responses. Disrupted tyrosine metabolism causes behavioral abnormalities (e.g., hyperactivity), increasing predation risk. Perturbed arachidonic acid metabolism impairs skin barrier function, enhancing TCC absorption and exposure to other contaminants. Additionally, increased SVL (metamorphic asynchrony) may result from energy allocation trade-offs, reducing post-metamorphic survival. This chain of events highlights the interconnectedness of biological levels: TCC’s impact on gut microbiota is a proximal driver of metabolic dysfunction, which in turn manifests as phenotypic toxicity. Importantly, this interplay is concentration-dependent: low TCC concentrations (5 μg/L) induce mild microbial shifts and metabolic compensation, while high concentrations (45 μg/L) lead to severe dysbiosis, irreversible metabolic damage, and significant phenotypic impairment.

## 5 Conclusion

Using integrated morphological, microbiome, and metabolomic analyses, our findings demonstrated that environmentally relevant concentrations of TCC induced acute and chronic toxicity in *R. taihangensis* tadpoles. Adverse effects included impaired morphological development (reduced body condition score, increased snout-vent length), gut microbial dysbiosis (decreased diversity, altered abundances of Pseudomonadota, Fusobacteriota, *Cetobacterium*, *Citrobacter*), and systemic metabolic disruption (dysregulated inflammation, neuro-endocrine signaling, energy/nutrient pathways). Significant microbiota-metabolite correlations confirmed gut dysbiosis contributes to metabolic dysfunction. TCC induced significant toxicity at a low concentration of 5 μg/L, demonstrating the inadequacy of current environmental standards. The co-occurrence of TCC with other aquatic pollutants in aquatic environments necessitates an assessment of potential synergistic effects. Our findings provide critical mechanistic insights into TCC’s ecotoxicity in aquatic vertebrates, highlighting the urgency for stricter TCC regulatory controls and advanced wastewater treatment technologies to mitigate its environmental impact and protect aquatic ecosystems.

## Data Availability

The 16S rRNA sequence raw data from this study were deposited in the NCBI SRA database (accession number PRJNA1347208). The metabolomic data are available upon request from the corresponding authors.

## Supplementary material

SUPPLEMENTARY FIGURE S1 Cumulative mortality (%) of *R. taihangensis* tadpoles after acute exposure to various concentrations of TCC in the acute toxicity assay.

SUPPLEMENTARY FIGURE S2 Kruskal–Wallis test and Dunn’s *post hoc* test were used to examine the differences in the solvent control group and TCC exposure groups at the phylum level **(a)** and genus level **(b)**. Only taxa with average relative abundances >0.01% were plotted. ^*^*p* < 0.05 and ^**^*p* < 0.01. CS, solvent control group; TL, 5 μg/L group; TM, 15 μg/L group; TH, 45 μg/L group.

SUPPLEMENTARY FIGURE S3 Differential metabolites between the solvent control group and the TCC treatment groups. ^*^*p* < 0.05, ^**^*p* < 0.01, and ^***^*p* < 0.001. CS, solvent control group; TL, 5 μg/L group; TM, 15 μg/L group; TH, 45 μg/L group.
