# Supplementary figures and images for: Triclocarban exposure at environmentally relevant concentrations perturbs the gut microbiota and metabolic profile in Rana taihangensis (Anura, Ranidae) tadpoles
Source: Front Microbiol. 2025 Dec 15;16:1740880. doi: 10.3389/fmicb.2025.1740880 (PMC12747674; doi:10.3389/fmicb.2025.1740880)

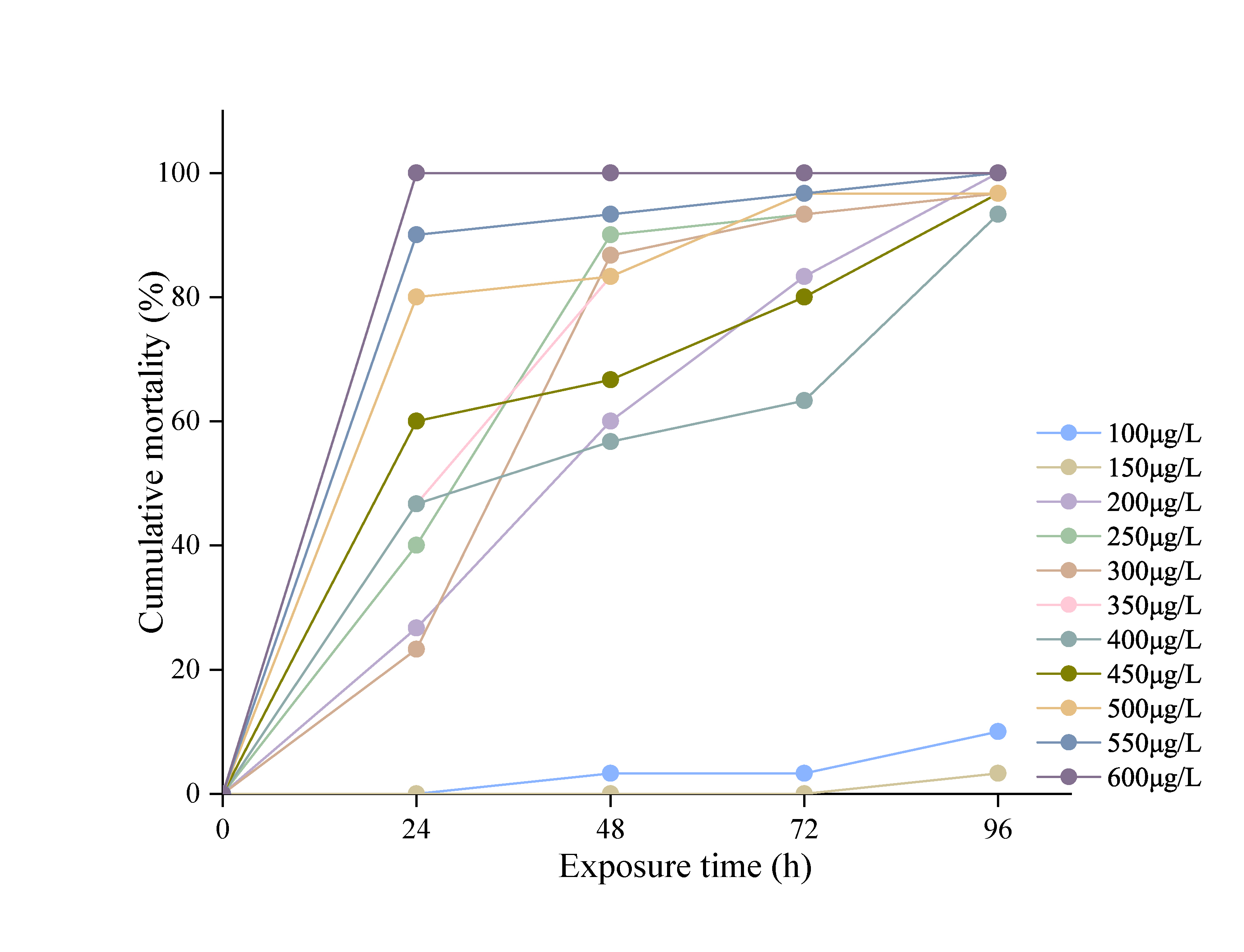

Supplement: SUPPLEMENTARY FIGURE S1 — Cumulative mortality (%) of R. taihangensis tadpoles after acute exposure to various concentrations of TCC in the acute toxicity assay. [file Image_1.JPEG]

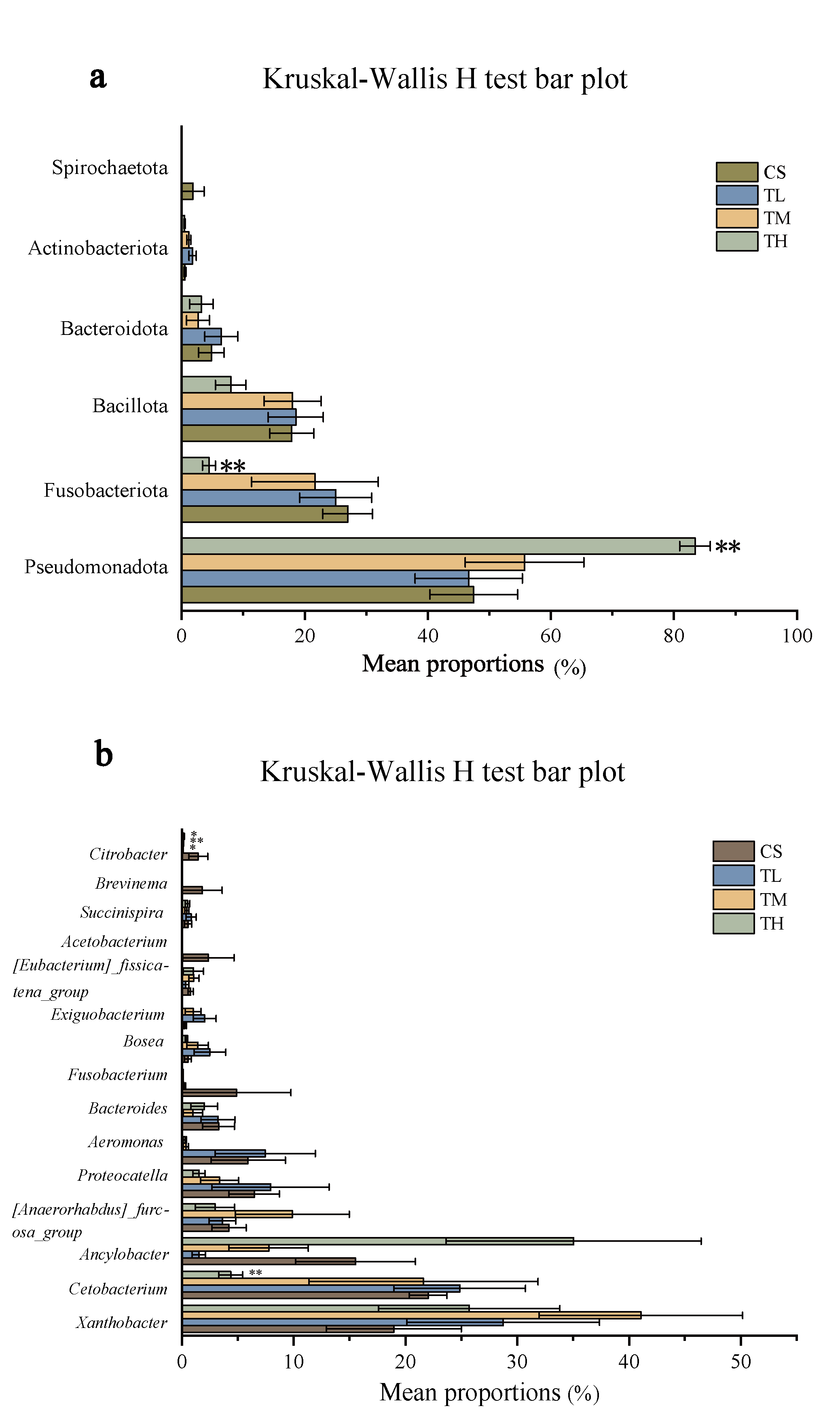

Supplement: SUPPLEMENTARY FIGURE S2 — Kruskal–Wallis test and Dunn’s post hoc test were used to examine the differences in the solvent control group and TCC exposure groups at the phylum level (a) and genus level (b). Only taxa with average relative abundances >0.01% were plotted. *p < 0.05 and **p < 0.01. CS, solvent control group; TL, 5 μg/L group; TM, 15 μg/L group; TH, 45 μg/L group. [file Image_2.JPEG]

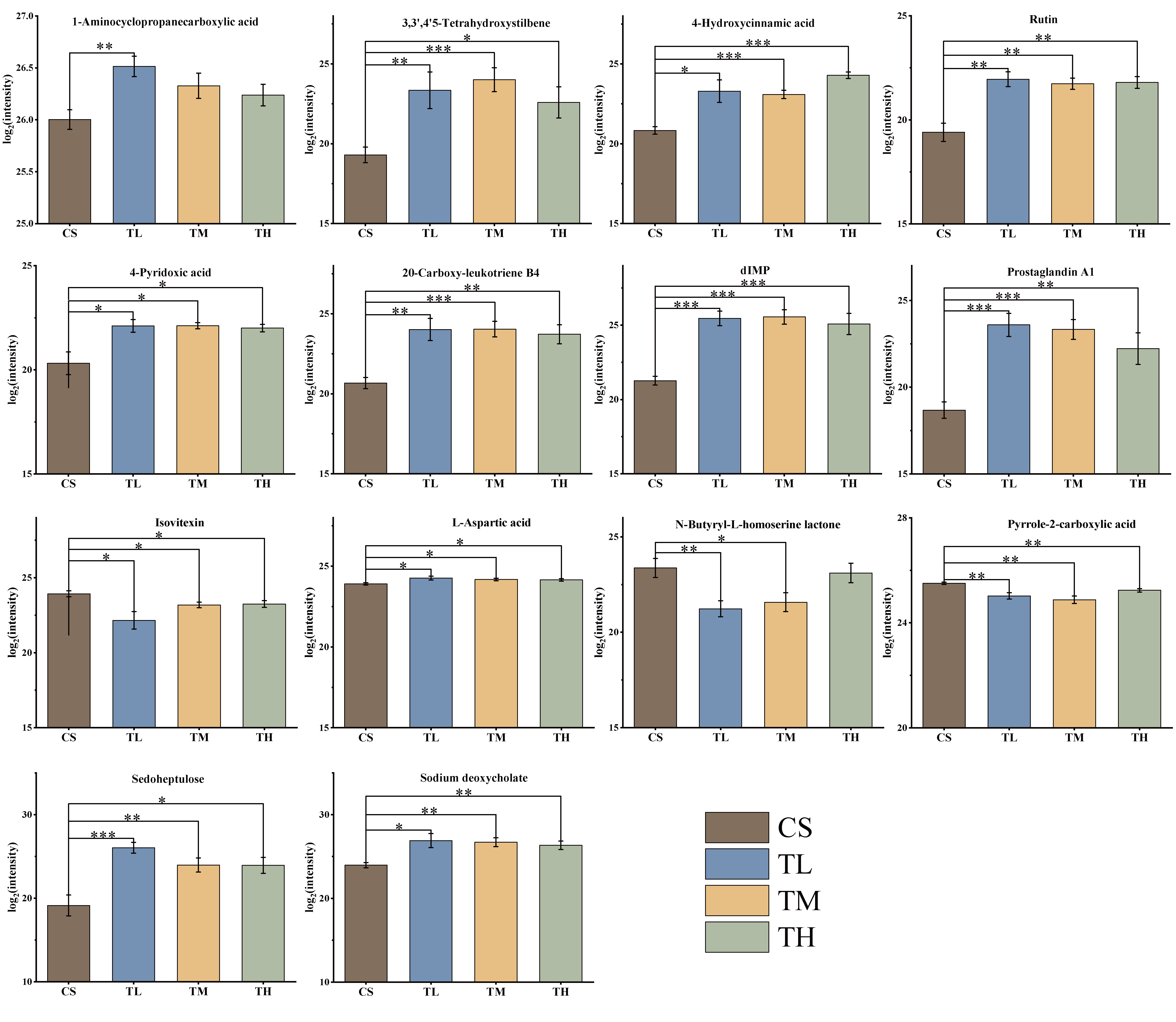

Supplement: SUPPLEMENTARY FIGURE S3 — Differential metabolites between the solvent control group and the TCC treatment groups. *p < 0.05, **p < 0.01, and ***p < 0.001. CS, solvent control group; TL, 5 μg/L group; TM, 15 μg/L group; TH, 45 μg/L group. [file Image_3.JPEG]
