# Supplementary material for: Triclocarban exposure at environmentally relevant concentrations perturbs the gut microbiota and metabolic profile in Rana taihangensis (Anura, Ranidae) tadpoles
Source: Front Microbiol. 2025 Dec 15;16:1740880. doi: 10.3389/fmicb.2025.1740880 (PMC12747674; doi:10.3389/fmicb.2025.1740880)
Supplement: Supplementary file 4 [file Table_1.docx]

SUPPLEMENTARY MATERIAL

**Table S1** In the forced exposure system, TCC concentration acted on the death data of *Rana taihanggensis* tadpoles at 24h, 48h, 72h and 96h.

|  | **Total exposed** | **24HRS** | **48HRS** | **72HRS** | **96HRS** |
| --- | --- | --- | --- | --- | --- |
| Solven control | 10 | 0 | 0 | 0 | 0 |
|  | 10 | 0 | 0 | 0 | 0 |
|  | 10 | 0 | 0 | 0 | 0 |
| 100 µg/L | 10 | 0 | 1 | 1 | 1 |
|  | 10 | 0 | 0 | 0 | 1 |
|  | 10 | 0 | 0 | 0 | 1 |
| 150 µg/L | 10 | 0 | 0 | 0 | 1 |
|  | 10 | 0 | 0 | 0 | 0 |
|  | 10 | 0 | 0 | 0 | 0 |
| 200 µg/L | 10 | 2 | 4 | 7 | 10 |
|  | 10 | 3 | 9 | 10 | — |
|  | 10 | 3 | 5 | 8 | 10 |
| 250 µg/L | 10 | 9 | 9 | 9 | 10 |
|  | 10 | 1 | 8 | 9 | 9 |
|  | 10 | 2 | 10 | — | — |
| 300 µg/L | 10 | 6 | 10 | — | — |
|  | 10 | 1 | 7 | 9 | 9 |
|  | 10 | 0 | 9 | 9 | 10 |
| 350 µg/L | 10 | 8 | 10 | — | — |
|  | 10 | 6 | 10 | — | — |
|  | 10 | 0 | 5 | 9 | 9 |
| 400 µg/L | 10 | 7 | 9 | 9 | 9 |
|  | 10 | 7 | 8 | 8 | 10 |
|  | 10 | 0 | 0 | 2 | 7 |
| 450 µg/L | 10 | 6 | 7 | 8 | 10 |
|  | 10 | 5 | 6 | 9 | 10 |
|  | 10 | 7 | 7 | 7 | 9 |
| 500 µg/L | 10 | 4 | 5 | 9 | 9 |
|  | 10 | 10 | — | — | — |
|  | 10 | 10 | — | — | — |
| 550 µg/L | 10 | 8 | 9 | 10 | — |
|  | 10 | 9 | 9 | 9 | 10 |
|  | 10 | 10 | — | — | — |
| 600 µg/L | 10 | 10 | — | — | — |
|  | 10 | 10 | — | — | — |
|  | 10 | 10 | — | — | — |

**Table S2** In the forced exposure system,77 metabolites overlapped in the three comparison groups. According to the HMDB database, the metabolites were classified by Super Class, and the metabolites that could not be retrieved in the database were labeled as others.

| **Metabolities** | **Super Class** | **CS vs TL** | | **CS vs TM** | | **CS vs TH** | |
| --- | --- | --- | --- | --- | --- | --- | --- |
|  |  | **VIP** | **P-value** | **VIP** | **P-value** | **VIP** | **P-value** |
| 4-Methoxybenzaldehyde | Benzenoids | 1.18 | 0.03 | 1.25 | 0.01 | 1.52 | <0.01 |
| Midodrine | Benzenoids | 1.44 | 0.01 | 1.38 | 0.01 | 1.28 | 0.02 |
| Tebuconazole | Benzenoids | 1.48 | <0.01 | 1.59 | <0.01 | 1.59 | <0.01 |
| (R)-Mandelamide | Benzenoids | 1.37 | 0.01 | 1.33 | 0.01 | 1.32 | 0.02 |
| 3-Hydroxyphenylacetic acid | Benzenoids | 1.37 | 0.03 | 1.29 | 0.05 | 1.51 | 0.01 |
| 3-Methoxy-4-hydroxyphenylglycolaldehyde | Benzenoids | 1.35 | 0.02 | 1.45 | 0.02 | 1.25 | 0.02 |
| Vanillylmandelic acid | Benzenoids | 1.25 | 0.03 | 1.24 | 0.02 | 1.19 | 0.04 |
| Norepinephrine | Benzenoids | 1.47 | 0.01 | 1.42 | 0.02 | 1.25 | 0.04 |
| Mibefradil | Benzenoids | 1.43 | 0.02 | 1.69 | <0.01 | 1.30 | 0.04 |
| Pyrophosphate | Homogeneous non-metal compounds | 1.43 | 0.01 | 1.56 | <0.01 | 1.24 | 0.03 |
| 20-Carboxy-leukotriene B_4_ | Lipids and lipid-like molecules | 1.38 | 0.01 | 1.46 | <0.01 | 1.27 | 0.02 |
| 11b-PGF2a | Lipids and lipid-like molecules | 1.34 | 0.01 | 1.68 | <0.01 | 1.63 | <0.01 |
| 14,15-DiHETrE | Lipids and lipid-like molecules | 1.50 | <0.01 | 1.54 | <0.01 | 1.40 | 0.01 |
| Azelaic acid | Lipids and lipid-like molecules | 1.60 | <0.01 | 1.68 | <0.01 | 1.61 | <0.01 |
| Leukotriene E_4_ | Lipids and lipid-like molecules | 1.34 | 0.03 | 1.27 | 0.03 | 1.31 | 0.02 |
| 16(R)-HETE | Lipids and lipid-like molecules | 1.15 | 0.05 | 1.46 | <0.01 | 1.20 | 0.04 |
| Prostaglandin A1 | Lipids and lipid-like molecules | 1.42 | 0.01 | 1.41 | 0.01 | 1.24 | 0.03 |
| Alpha-dimorphecolic acid | Lipids and lipid-like molecules | 1.68 | <0.01 | 1.37 | 0.01 | 1.52 | <0.01 |
| Hepoxilin B_3_ | Lipids and lipid-like molecules | 1.21 | 0.04 | 1.58 | <0.01 | 1.54 | <0.01 |
| Isovitexin | Lipids and lipid-like molecules | 1.28 | 0.01 | 1.26 | 0.03 | 1.15 | 0.04 |
| Mesaconate | Lipids and lipid-like molecules | 1.20 | 0.04 | 1.15 | 0.04 | 1.31 | 0.02 |
| alpha-Tocopherol | Lipids and lipid-like molecules | 1.44 | 0.01 | 1.41 | 0.01 | 1.66 | <0.01 |
| Androstanedione | Lipids and lipid-like molecules | 1.40 | 0.01 | 1.40 | 0.01 | 1.47 | 0.01 |
| 17a-Estradiol | Lipids and lipid-like molecules | 1.76 | 0.00 | 1.74 | <0.01 | 1.46 | 0.01 |
| Cholesterol | Lipids and lipid-like molecules | 1.46 | 0.01 | 1.61 | <0.01 | 1.41 | 0.01 |
| Androsterone | Lipids and lipid-like molecules | 1.26 | 0.03 | 1.23 | 0.04 | 1.20 | 0.04 |
| Sodium deoxycholate | Lipids and lipid-like molecules | 1.33 | 0.03 | 1.26 | 0.02 | 1.19 | 0.05 |
| Adenosine | Nucleosides, nucleotides, and analogues | 1.37 | 0.01 | 1.31 | 0.02 | 1.30 | 0.02 |
| Xanthylic acid | Nucleosides, nucleotides, and analogues | 1.42 | 0.01 | 1.64 | <0.01 | 1.53 | <0.01 |
| dIMP | Nucleosides, nucleotides, and analogues | 1.29 | 0.02 | 1.37 | 0.01 | 1.42 | 0.01 |
| Orotidylic acid | Nucleosides, nucleotides, and analogues | 1.61 | <0.01 | 1.68 | <0.01 | 1.26 | 0.02 |
| Betaine | Organic acids and derivatives | 1.44 | 0.01 | 1.63 | <0.01 | 1.35 | 0.01 |
| L-2,4-diaminobutyric acid | Organic acids and derivatives | 1.49 | 0.01 | 1.52 | <0.01 | 1.30 | 0.02 |
| Racemethionine | Organic acids and derivatives | 1.40 | 0.01 | 1.60 | 0.00 | 1.34 | 0.02 |
| Glycylleucine | Organic acids and derivatives | 1.67 | <0.01 | 1.40 | 0.01 | 1.36 | 0.01 |
| L-Aspartic acid | Organic acids and derivatives | 1.35 | 0.03 | 1.18 | 0.04 | 1.24 | 0.04 |
| Kyotorphin | Organic acids and derivatives | 1.41 | 0.01 | 1.63 | <0.01 | 1.32 | 0.02 |
| Aspartame | Organic acids and derivatives | 1.21 | 0.03 | 1.21 | 0.04 | 1.30 | 0.02 |
| N6,N6,N6-Trimethyl-L-lysine | Organic acids and derivatives | 1.29 | 0.02 | 1.37 | 0.01 | 1.57 | <0.01 |
| Asymmetric dimethylarginine | Organic acids and derivatives | 1.25 | 0.02 | 1.29 | 0.01 | 1.35 | 0.01 |
| (5-L-Glutamyl)-L-glutamate | Organic acids and derivatives | 1.71 | <0.01 | 1.43 | 0.01 | 1.62 | <0.01 |
| Citrulline | Organic acids and derivatives | 1.39 | 0.01 | 1.54 | <0.01 | 1.56 | <0.01 |
| L-Lysine | Organic acids and derivatives | 1.43 | 0.01 | 1.27 | 0.02 | 1.16 | 0.04 |
| L-Phenylalanine | Organic acids and derivatives | 1.42 | 0.02 | 1.58 | <0.01 | 1.57 | <0.01 |
| D-Lysopine | Organic acids and derivatives | 1.45 | 0.01 | 1.50 | <0.01 | 1.47 | <0.01 |
| 2-Hydroxyglutarate | Organic acids and derivatives | 1.49 | <0.01 | 1.52 | <0.01 | 1.51 | <0.01 |
| Acetylphosphate | Organic acids and derivatives | 1.24 | 0.03 | 1.37 | 0.01 | 1.45 | 0.01 |
| Sphingosine | Organic nitrogen compounds | 1.42 | 0.01 | 1.28 | 0.02 | 1.24 | 0.03 |
| L-3-Hydroxykynurenine | Organic oxygen compounds | 1.39 | 0.01 | 1.68 | <0.01 | 1.84 | <0.01 |
| 2,3-Butanediol | Organic oxygen compounds | 1.81 | <0.01 | 1.77 | <0.01 | 1.67 | <0.01 |
| Sedoheptulose | Organic oxygen compounds | 1.41 | 0.02 | 1.36 | 0.02 | 1.32 | 0.03 |
| N-Acetyl-a-neuraminic acid | Organic oxygen compounds | 1.21 | 0.02 | 1.35 | 0.01 | 1.28 | 0.02 |
| Imidazolepropionic acid | Organoheterocyclic compounds | 1.53 | <0.01 | 1.19 | 0.04 | 1.51 | <0.01 |
| 10-Hydroxycarbazepine | Organoheterocyclic compounds | 1.25 | 0.04 | 1.35 | 0.02 | 1.15 | 0.05 |
| 2-Furoate | Organoheterocyclic compounds | 1.44 | 0.02 | 1.74 | <0.01 | 1.87 | <0.01 |
| D-Glucurono-6,3-lactone | Organoheterocyclic compounds | 1.61 | <0.01 | 1.48 | 0.01 | 1.50 | 0.01 |
| Guanine | Organoheterocyclic compounds | 1.53 | <0.01 | 1.64 | <0.01 | 1.50 | 0.01 |
| Hypoxanthine | Organoheterocyclic compounds | 1.19 | 0.04 | 1.56 | <0.01 | 1.28 | 0.02 |
| Pindolol | Organoheterocyclic compounds | 1.23 | 0.04 | 1.21 | 0.03 | 1.42 | 0.01 |
| 5-Hydroxyindoleacetic acid | Organoheterocyclic compounds | 1.44 | 0.01 | 1.42 | 0.01 | 1.27 | 0.02 |
| Isoquinoline | Organoheterocyclic compounds | 1.20 | 0.01 | 1.41 | 0.01 | 1.32 | 0.02 |
| Lumichrome | Organoheterocyclic compounds | 1.20 | 0.03 | 1.27 | 0.02 | 1.88 | <0.01 |
| 4-Pyridoxic acid | Organoheterocyclic compounds | 1.60 | <0.01 | 1.68 | <0.01 | 1.53 | <0.01 |
| Nicotinic acid | Organoheterocyclic compounds | 1.26 | 0.02 | 1.55 | <0.01 | 1.23 | 0.04 |
| Pyrrole-2-carboxylic acid | Organoheterocyclic compounds | 1.30 | 0.03 | 1.42 | 0.01 | 1.44 | 0.02 |
| 4-Quinolinecarboxylic acid | Organoheterocyclic compounds | 1.01 | 0.04 | 1.44 | <0.01 | 1.20 | 0.03 |
| 4-Hydroxycinnamic acid | Phenylpropanoids and polyketides | 1.53 | 0.01 | 1.57 | 0.01 | 1.45 | 0.01 |
| trans-Cinnamate | Phenylpropanoids and polyketides | 1.42 | 0.01 | 1.65 | <0.01 | 1.36 | 0.01 |
| Hecogenin | Phenylpropanoids and polyketides | 1.41 | <0.01 | 1.54 | <0.01 | 1.48 | 0.01 |
| Rutin | Phenylpropanoids and polyketides | 1.39 | 0.01 | 1.38 | 0.01 | 1.28 | 0.02 |
| 3,3',4'5-Tetrahydroxystilbene | Phenylpropanoids and polyketides | 1.48 | 0.02 | 1.53 | 0.01 | 1.26 | 0.05 |
| 9,10-Dihydroxy-12,13-epoxyoctadecanoate | Others | 1.45 | 0.01 | 1.44 | 0.01 | 1.29 | 0.02 |
| (5Z,9E,14Z)-(8xi,11xi,12S)-8,11,12-Trihydroxyicosa-5,9,14-trienoate | Others | 1.19 | 0.04 | 1.23 | 0.03 | 1.38 | 0.01 |
| Nopaline | Others | 1.41 | 0.01 | 1.50 | 0.01 | 1.20 | 0.04 |
| 8,12-Diethyl-3-vinylbacteriochlorophyllide d | Others | 1.28 | 0.04 | 1.42 | 0.01 | 1.53 | <0.01 |
| Cannabielsoin | Others | 1.57 | <0.01 | 1.47 | <0.01 | 1.42 | 0.01 |
| Cyclopeptine | Others | 1.64 | <0.01 | 1.57 | <0.01 | 1.64 | <0.01 |

**Table S3** Based on the 77 common differential metabolites, KEGG was enriched into 21 significantly different metabolic pathways.

| **Metabolism pathway** | | | **P-value** | **Hits** |
| --- | --- | --- | --- | --- |
| **Description** | **level1** | **level2** |  |  |
| Arachidonic acid metabolism | Metabolism | Lipid metabolism | <0.001 | 6 |
| Tyrosine metabolism | Metabolism | Amino acid metabolism | 0.002 | 5 |
| Neuroactive ligand-receptor interaction | Environmental Information Processing | Signaling molecules and interaction | 0.004 | 4 |
| ABC transporters | Environmental Information Processing | Membrane transport | 0.006 | 6 |
| Purine metabolism | Metabolism | Nucleotide metabolism | 0.008 | 5 |
| D-Amino acid metabolism | Metabolism | Metabolism of other amino acids | 0.010 | 4 |
| Regulation of lipolysis in adipocytes | Organismal Systems | Endocrine system | 0.012 | 2 |
| Sphingolipid signaling pathway | Environmental Information Processing | Signal transduction | 0.014 | 2 |
| Vascular smooth muscle contraction | Organismal Systems | Circulatory system | 0.016 | 2 |
| Renin secretion | Organismal Systems | Endocrine system | 0.018 | 2 |
| Protein digestion and absorption | Organismal Systems | Digestive system | 0.019 | 3 |
| Biosynthesis of amino acids | Metabolism | Global and overview maps | 0.020 | 5 |
| Glycine, serine and threonine metabolism | Metabolism | Amino acid metabolism | 0.021 | 3 |
| Phenylalanine metabolism | Metabolism | Amino acid metabolism | 0.022 | 3 |
| Aminoacyl-tRNA biosynthesis | Genetic Information Processing | Translation | 0.025 | 3 |
| Lysine degradation | Metabolism | Amino acid metabolism | 0.031 | 3 |
| Arginine biosynthesis | Metabolism | Amino acid metabolism | 0.032 | 2 |
| Steroid hormone biosynthesis | Metabolism | Lipid metabolism | 0.032 | 4 |
| cAMP signaling pathway | Environmental Information Processing | Signal transduction | 0.037 | 2 |
| Linoleic acid metabolism | Metabolism | Lipid metabolism | 0.046 | 2 |
| Apoptosis | Cellular Processes | Cell growth and death | 0.048 | 1 |

**Table S4** A total of 23 metabolites were enriched in 5 metabolic pathways selected according to the conditions ( *p* < 0.05 and Hits ≥ 5 ).

| **Metabolism pathway** | **Metabolities** |
| --- | --- |
| Arachidonic acid metabolism | 16(R)-HETE; 11b-PGF2a; 14,15-DiHETrE; Hepoxilin B_3_; 20-Carboxy-leukotriene B_4_; Leukotriene E_4_ |
| ABC transporters | Betaine; L-Lysine; L-Phenylalanine; L-Aspartic acid; Adenosine; Nopaline |
| Purine metabolism | Hypoxanthine; dIMP; Guanine; Adenosine; Xanthylic acid |
| Biosynthesis of amino acids | L-Lysine; L-Phenylalanine; Citrulline; L-Aspartic acid; L-2,4-diaminobutyric acid |
| Tyrosine metabolism | 3-Methoxy-4-hydroxyphenylglycolaldehyde; 4-Hydroxycinnamic acid; Vanillylmandelic acid; 3-Hydroxyphenylacetic acid; Norepinephrine |

**Table S5** A total of 86 metabolic markers were screened by LEfSe analysis. According to the HMDB database, the metabolites were classified by Super Class, and the metabolites that could not be retrieved in the database were labeled as others.

| **metabolic marker** | **Super Class** | **Groups** | **LDA_values** | **P-value** |
| --- | --- | --- | --- | --- |
| Harmaline | Alkaloids and derivatives | CS | 2.78 | 0.003 |
| 2-Phenylethanol | Benzenoids | CS | 2.75 | 0.003 |
| 3-Hydroxyphenylacetic acid | Benzenoids | TM | 2.39 | 0.021 |
| 3-Methoxy-4-hydroxyphenylglycolaldehyde | Benzenoids | CS | 2.70 | 0.007 |
| Aminocarb | Benzenoids | CS | 2.63 | 0.007 |
| Dulcin | Benzenoids | CS | 2.80 | 0.016 |
| Epinephrine | Benzenoids | CS | 2.75 | 0.003 |
| Folic acid | Benzenoids | CS | 2.57 | 0.008 |
| Methyleugenol | Benzenoids | CS | 2.40 | 0.004 |
| Mibefradil | Benzenoids | TM | 4.04 | 0.044 |
| Midodrine | Benzenoids | TM | 2.44 | 0.039 |
| Oxymorphone | Benzenoids | CS | 2.49 | 0.012 |
| Propoxur | Benzenoids | CS | 2.58 | 0.005 |
| 11b-PGF2a | Lipids and lipid-like molecules | TM | 3.05 | 0.010 |
| 17a-Estradiol | Lipids and lipid-like molecules | TL | 2.61 | 0.027 |
| 20a,22b-Dihydroxycholesterol | Lipids and lipid-like molecules | CS | 2.63 | 0.014 |
| 20-Carboxy-leukotriene B4 | Lipids and lipid-like molecules | TL | 2.48 | 0.015 |
| 4a-Carboxy-4b-methyl-5a-cholesta-8,24-dien-3b-ol | Lipids and lipid-like molecules | CS | 2.52 | 0.005 |
| 9(S)-HPODE | Lipids and lipid-like molecules | CS | 2.70 | 0.003 |
| 9,10-Dihydroxystearate | Lipids and lipid-like molecules | CS | 2.45 | 0.035 |
| 9-OxoODE | Lipids and lipid-like molecules | CS | 3.09 | 0.004 |
| alpha-Tocopherol | Lipids and lipid-like molecules | TM | 2.43 | 0.049 |
| Androsta-1,4-diene-3,17-dione | Lipids and lipid-like molecules | CS | 2.56 | 0.011 |
| Androstanedione | Lipids and lipid-like molecules | TH | 3.06 | 0.028 |
| Arachidic acid | Lipids and lipid-like molecules | CS | 3.46 | 0.032 |
| Azelaic acid | Lipids and lipid-like molecules | CS | 2.38 | 0.014 |
| Chenodeoxycholic acid | Lipids and lipid-like molecules | TH | 2.86 | 0.005 |
| Cholesterol | Lipids and lipid-like molecules | TM | 2.44 | 0.029 |
| Isovitexin | Lipids and lipid-like molecules | CS | 2.61 | 0.006 |
| Lutein | Lipids and lipid-like molecules | CS | 3.32 | 0.004 |
| Prostaglandin A1 | Lipids and lipid-like molecules | TL | 2.51 | 0.006 |
| Prostaglandin B2 | Lipids and lipid-like molecules | CS | 2.67 | 0.005 |
| Sodium deoxycholate | Lipids and lipid-like molecules | TL | 3.28 | 0.023 |
| Stearic acid | Lipids and lipid-like molecules | CS | 3.79 | 0.006 |
| Ubiquinone-1 | Lipids and lipid-like molecules | CS | 2.53 | 0.006 |
| Vaccenic acid | Lipids and lipid-like molecules | CS | 3.15 | 0.002 |
| Deoxycytidine | Nucleosides, nucleotides, and analogues | TL | 2.81 | 0.033 |
| Deoxyuridine-5'-triphosphate | Nucleosides, nucleotides, and analogues | TL | 3.37 | 0.049 |
| dGMP | Nucleosides, nucleotides, and analogues | CS | 4.08 | 0.023 |
| dIMP | Nucleosides, nucleotides, and analogues | TM | 2.92 | 0.005 |
| dTMP | Nucleosides, nucleotides, and analogues | CS | 2.51 | 0.032 |
| Orotidylic acid | Nucleosides, nucleotides, and analogues | CS | 2.40 | 0.036 |
| Adenosine | Nucleosides,nucleotides, and analogues | TM | 2.39 | 0.016 |
| (S)-5-Amino-3-oxohexanoate | Organic acids and derivatives | CS | 2.70 | 0.011 |
| 1-Aminocyclopropanecarboxylic acid | Organic acids and derivatives | CS | 2.73 | 0.010 |
| Acetylphosphate | Organic acids and derivatives | CS | 2.82 | 0.002 |
| alpha-Ketoisovaleric acid | Organic acids and derivatives | CS | 2.58 | 0.007 |
| D-Octopine | Organic acids and derivatives | CS | 2.68 | 0.010 |
| L-2,4-diaminobutyric acid | Organic acids and derivatives | CS | 2.32 | 0.024 |
| L-Aspartic acid | Organic acids and derivatives | CS | 2.45 | 0.005 |
| L-Threonine | Organic acids and derivatives | CS | 3.43 | 0.027 |
| N6-Acetyl-L-lysine | Organic acids and derivatives | CS | 3.64 | 0.011 |
| N-Butyryl-L-homoserine lactone | Organic acids and derivatives | CS | 2.62 | 0.007 |
| Pyrrolidonecarboxylic acid | Organic acids and derivatives | CS | 2.74 | 0.002 |
| Tropate | Organic acids and derivatives | CS | 4.58 | 0.003 |
| Ureidosuccinic acid | Organic acids and derivatives | CS | 2.74 | 0.003 |
| 1-Methylhistamine | Organic nitrogen compounds | CS | 2.76 | 0.006 |
| Sphinganine | Organic nitrogen compounds | CS | 2.43 | 0.005 |
| 2,3-Butanediol | Organic oxygen compounds | CS | 2.90 | 0.010 |
| 3-Hydroxybenzyl alcohol glucoside | Organic oxygen compounds | CS | 3.43 | 0.009 |
| Glucosamine 6-phosphate | Organic oxygen compounds | TH | 3.34 | 0.002 |
| L-3-Hydroxykynurenine | Organic oxygen compounds | TM | 2.20 | 0.020 |
| Sedoheptulose | Organic oxygen compounds | TL | 3.10 | 0.013 |
| Sucrose | Organic oxygen compounds | CS | 2.34 | 0.040 |
| 10-Hydroxycarbazepine | Organoheterocyclic compounds | CS | 2.52 | 0.012 |
| 4-Pyridoxic acid | Organoheterocyclic compounds | TM | 2.67 | 0.039 |
| 5-(2-Hydroxyethyl)-4-methylthiazole | Organoheterocyclic compounds | CS | 2.51 | 0.004 |
| 7-Methylguanine | Organoheterocyclic compounds | TL | 4.33 | 0.036 |
| Guanine | Organoheterocyclic compounds | TH | 2.68 | 0.041 |
| Ipronidazole | Organoheterocyclic compounds | CS | 2.43 | 0.007 |
| N-Methyl-2-pyrrolidinone | Organoheterocyclic compounds | CS | 3.36 | 0.009 |
| Pyrrole-2-carboxylic acid | Organoheterocyclic compounds | CS | 2.79 | 0.004 |
| Tryptophanol | Organoheterocyclic compounds | CS | 2.80 | 0.018 |
| 3,3',4'5-Tetrahydroxystilbene | Phenylpropanoids and polyketides | TM | 2.51 | 0.021 |
| 4-Hydroxycinnamic acid | Phenylpropanoids and polyketides | TL | 2.38 | 0.008 |
| Hecogenin | Phenylpropanoids and polyketides | CS | 2.78 | 0.003 |
| Rutin | Phenylpropanoids and polyketides | TL | 2.73 | 0.042 |
| (6Z)-Octadecenoic acid | other | TH | 3.15 | 0.005 |
| 9,10-Dihydroxy-12,13-epoxyoctadecanoate | other | TL | 2.86 | 0.013 |
| Andrographolide | other | CS | 2.65 | 0.005 |
| Coleonol | other | TH | 2.35 | 0.032 |
| Cyclopeptine | other | TL | 2.41 | 0.034 |
| Farnesoic acid | other | CS | 2.41 | 0.035 |
| L-N2-(2-Carboxyethyl)arginine | other | CS | 2.79 | 0.002 |
| N5-(L-1-Carboxyethyl)-L-ornithine | other | TL | 2.39 | 0.037 |
| Scopoline | other | CS | 2.53 | 0.003 |

**Table S6** Spearman correlation analysis identified 15 pairs of microbial markers and metabolic markers with a strong correlation (|rho| > 0.7).

| **microbial marker** | **metabolic marker** | **rho** | **P-value** | **relation** |
| --- | --- | --- | --- | --- |
| *Lactobacillus* | Isovitexin | -0.76 | < 0.001 | negative |
| *Lactobacillus* | N-Butyryl-L-homoserine lactone | -0.70 | < 0.001 | negative |
| *Limosilactobacillus* | Pyrrole-2-carboxylic acid | -0.74 | < 0.001 | negative |
| *Lactobacillus* | 1-Aminocyclopropanecarboxylic acid | 0.71 | < 0.001 | positive |
| *Lactobacillus* | Prostaglandin A1 | 0.72 | < 0.001 | positive |
| *Lactobacillus* | Sedoheptulose | 0.73 | < 0.001 | positive |
| *Limosilactobacillus* | 3,3',4'5-Tetrahydroxystilbene | 0.72 | < 0.001 | positive |
| *Limosilactobacillus* | 4-Pyridoxic acid | 0.72 | < 0.001 | positive |
| *Limosilactobacillus* | L-Aspartic acid | 0.72 | < 0.001 | positive |
| *Limosilactobacillus* | Rutin | 0.72 | < 0.001 | positive |
| *Limosilactobacillus* | dIMP | 0.73 | < 0.001 | positive |
| *Limosilactobacillus* | Sodium deoxycholate | 0.74 | < 0.001 | positive |
| *Limosilactobacillus* | 1-Aminocyclopropanecarboxylic acid | 0.76 | < 0.001 | positive |
| *Limosilactobacillus* | 20-Carboxy-leukotriene B4 | 0.76 | < 0.001 | positive |
| *Thiobacillus* | 4-Hydroxycinnamic acid | 0.71 | < 0.001 | positive |
